# Parent-Mediated Interventions for Children and Adolescents With Autism Spectrum Disorders: A Systematic Review and Meta-Analysis

**DOI:** 10.3389/fpsyt.2021.773604

**Published:** 2021-11-12

**Authors:** Charlotte Engberg Conrad, Marie Louise Rimestad, Jeanett Friis Rohde, Birgitte Holm Petersen, Christoffer Bruun Korfitsen, Simon Tarp, Cathriona Cantio, Marlene Briciet Lauritsen, Mina Nicole Händel

**Affiliations:** ^1^Psychiatry, Department of Clinical Medicine, Aalborg University Hospital, Aalborg University, Aalborg, Denmark; ^2^Institute of Psychology, Aarhus University, Aarhus, Denmark; ^3^The Parker Institute, Bispebjerg and Frederiksberg Hospital, The Capital Region, Frederiksberg, Denmark; ^4^The Danish Health Authority, Copenhagen, Denmark; ^5^Institute of Psychology, University of Southern Denmark, Odense, Denmark; ^6^Child and Adolescent Mental Health Services, Region of Southern Denmark, Odense, Denmark

**Keywords:** autistic disorder, autism spectrum disorder, parent-mediated intervention, caregiver-mediated intervention, early intervention, treatment outcome

## Abstract

There has been increasing interest in parent-mediated interventions (PMIs) for children with autism spectrum disorders (ASDs). The objective of this systematic review and meta-analysis was to examine the effect of PMIs compared to no PMI for children with ASD aged 2–17 years. The primary outcome was adaptive functioning rated by a parent or clinician. The secondary outcomes were long-term adaptive functioning rated by the parents, adverse events, core symptoms of ASD, disruptive behavior, parental well-being, quality of life of the child rated by the parents and anxiety. The MEDLINE, PsycInfo, Embase, and CINAHL databases were searched in March 2020. The Cochrane Risk of Bias Tool was used to rate the individual studies, and the certainty in the evidence was evaluated using GRADE. We identified 30 relevant randomized controlled trials (RCTs), including 1,934 participants. A clinically relevant effect of PMIs on parent-rated adaptive functioning was found with a low certainty of evidence [Standard mean difference (SMD): 0.28 (95% CI: −0.01, 0.57)] on Vineland Adaptive Behavior Scales (VABS), whereas no clinically relevant effect was seen for clinician-rated functional level, with a very low certainty of evidence [SMD on Clinical Global Impressions (CGI)-severity scale: SMD −0.45 [95% CI: −0.87, −0.03)]. PMIs may slightly improve clinician-rated autism core symptoms [SMD: −0.35 (95% CI: −0.71, 0.02)]. Additionally, no effect of PMIs on parent-rated core symptoms of ASD, parental well-being or adverse effects was identified, all with a low certainty of evidence. There was a moderate certainty of evidence for a clinically relevant effect on disruptive behavior [SMD: 0.55 (95% Cl: 0.36, 0.74)]. The certainty in the evidence was downgraded due to serious risk of bias, lack of blinding, and serious risk of imprecision due to few participants included in meta-analyses. The present findings suggest that clinicians may consider introducing PMIs to children with ASD, but more high-quality RCTs are needed because the effects are not well-established, and the results are likely to change with future studies. The protocol for the systematic review is registered at the Danish Health Authority website (www.sst.dk).

## Introduction

Autism spectrum disorder (ASD) is a neurodevelopmental disorder characterized by early-onset difficulties in social interaction, communication and stereotyped repetitive behaviors and interests (1, 2), and it affects ~1% of the population in high-income countries (3). The disorder is expected to be lifelong, and people on the spectrum are reported to have an elevated mortality risk, lower educational level, reduced quality of life and higher frequency of comorbid disorders, e.g., depression and anxiety (2, 4–6).

Various behavioral and structural interventions have been developed to improve adaptive functioning, behavioral problems and quality of life for people with ASD, and some interventions reduce ASD symptomatology (2, 7, 8). Several early behavioral interventions have also been developed (9). In recent decades, increasing interest in early parent-child interactions has been observed, the involvement of parents in therapy has increased, and various parent-mediated interventions (PMIs) have been developed.

PMIs are advantageous because they reduce the demands on children with ASD compared to behavioral approaches and bring treatment into a home and community setting, enabling the transfer of skills to real-life settings. PMIs engage parents in the role of a therapist to implement interventions in an individualized and sensitive way (10). Parents already spend much time with their children, which provides an obvious opportunity for a cost-effective intervention, with extensive implementation and generalization opportunities in everyday life and through different contexts (11).

The PMIs vary, including teaching comprehensive skills and others targeting specific impairments, e.g., joint attention, communication, or language (2). The various PMIs cover education, training and coaching from clinicians to parents, with the overarching aim of improving opportunities for children to learn through different contexts. This is essential to ensure generalization and maintain treatment gains (11, 12). Initially, PMIs targeted younger children with ASD; however, simultaneously with the broadening of treatment targets, the age group has broadened to include subjects from early childhood into young adulthood (11).

Several reviews with or without meta-analysis have been conducted in the research field (10, 13–16), but only a few have used Grades of Recommendation, Assessment, Development, and Evaluation (GRADE) to assess the certainty of the evidence (10, 13), and none have addressed both beneficial and adverse aspects of PMIs. The outcome focus of the reviews varies considerably, differing from child outcomes, such as language, adaptive functioning and social communication, to parent outcomes, such as parental stress and quality of life; only a few reviews included a meta-analysis (10, 13, 15–17). Some reviews expanded the parent concept to parent-focused interventions and included all interventions that included parents (17).

A more recent systematic review and meta-analysis of outcomes of PMIs for younger children by Nevill et al. (13) found moderate positive outcomes of PMIs regarding language communication, autism symptom severity, and cognition, but the evidence of positive changes in socialization was very low. The review concluded that the overall quality of more recent randomized controlled trials (RCTs) is improving (13). Several RCTs have been published since the Nevill et al. (13) review and meta-analysis. Thus, to provide clinicians and guideline panels as well as caregivers with an updated overview of the current evidence from RCTs, the objective of this systematic review and meta-analysis was to synthesize the effect of PMIs for children and adolescents with autism aged 18 months−17 years on both beneficial and adverse outcomes.

In particular the following questions were addressed:

- What is the overall effect of the PMIs for children and adolescents with autism?
- What is the effect of the PMIs on parental stress, parental well-being and quality of life?
- Which adverse effects of the PMIs are seen in current research?
- What is the quality of the identified research in this field?
- Will current evidence of the PMIs be sufficient to recommend PMIs for children with autism?

## Methods

This systematic review and meta-analysis was conducted according to the recommendations of the Cochrane Handbook (18) and is reported according to the Preferred Reporting Items for Systematic Reviews and Meta-Analyses (PRISMA) statement (19) (Prisma checklist is provided in Supplementary Table 1). The systematic review also follows the structure described by the population, intervention, comparison and outcome (PICO) characterization (20). The certainty in the evidence was rated by the GRADE approach (21). The study is part of the national Clinical Practice Guidelines on the treatment of ASD among children and adolescents published by the Danish Health Authority in 2021 (22). The content of the study protocol, including review question, search strategy, inclusion and exclusion criteria, and risk of bias assessment, was prespecified, registered and approved by the management at the Danish Health Authority in November 2019 (and the protocol is available at the Danish Health Authority website: www.sst.dk).

### Search Strategy

The systematic search was conducted in March 2020 by a search specialist (B.H.P.). The databases searched were MEDLINE, PsycInfo, Embase, and CINAHL. The search was performed in two steps: (a) search for systematic reviews and meta-analyses with a filter and (b) search for primary literature, both with a combination of medical subject heading (MeSH)/index terms and free-text searches. The search was limited to articles in English and Scandinavian language referring to language skills of the review authors. The detailed search strategy is presented in the Supplementary Table 2.

A cross reference search and screening of reference lists of included articles and previous reviews was performed, and the guideline working group members (content experts) conferred whether any studies were missing from the search. Study authors were not contacted to identify additional studies.

### Study Selection

Articles generated from the defined search strategy of individual RCTs were deduplicated and imported from RefWorks into Covidence software for literature screening and data management (www.covidence.org). According to prespecified PICO criteria, one reviewer (M.L.R.) evaluated the titles and abstracts of eligible articles (see below). The identified full-text articles were independently screened by two reviewers (review authors: M.L.R., C.C. and M.B.L.). Any disagreements were resolved through discussion. The eligible studies had to match the following criteria:

### Population

Children and adolescents from 18 months to 17 years of age diagnosed with ASD according to diagnostic criteria with or without comorbidities.

### Intervention

Parent-mediated interventions for children and adolescents with ASD with 8 or more sessions.

### Comparator

No parent-mediated intervention.

### Outcomes

#### Primary Outcome

Adaptive functioning was rated at a minimum of 8 weeks by the parent or clinician.

#### Secondary Outcomes

- Parent-rated adaptive functioning after at least 6 months of follow-up
- Adverse effects at minimum 8 weeks
- Core symptoms of ASD, parent- or clinician-rated at a minimum of 8 weeks
- Disruptive behavior, parent-rated at a minimum of 8 weeks
- Parental well-being at a minimum of 8 weeks
- Quality of life, parent-rated at a minimum of 8 weeks
- Anxiety at a minimum of 8 weeks

### Study Design

Only RCTs were included in this review. The aim of random assignment used in RCTs is to prevent selection bias by distributing the characteristics of patients who may influence the outcome randomly between the groups; therefore, RCTs are considered to minimize the risk of confounding factors influencing the results, thus providing the most reliable evidence on the effectiveness of interventions.

### Data Extraction of Individual Randomized Trials

A predefined template in Covidence software was used to conduct data extraction independently by two out of three reviewers (J.F.R., C.B.K., M.N.H.). Extraction of the following descriptive and quantitative characteristics was performed:

1. Characteristics of the study: authorship, year, country, setting, sample size, design, methods, duration of follow-up, source of funding, conflict of interest.
2. Characteristics of the population: age, race/ethnicity, socioeconomic status, cointerventions, information regarding respondent bias, or representativeness of the included population.
3. Description of the intervention.
4. Description of the comparator group.
5. Outcomes and timepoints for outcomes, as mentioned above.

### Risk of Bias and Certainty of Evidence

We used GRADE to assess the certainty of evidence, which was categorized as very low, low, moderate, and high (21). Each RCT study started at a high certainty level and was assessed for possibly being rated down based on five domains: overall risk of bias, inconsistency, indirectness, imprecision, and publication bias. Following GRADE, whenever sample size in the analysis were <100 participants, the study was downgraded for imprecision.

The criterion provided by the Cochrane Collaboration's tool for assessing the risk of bias of RCTs (18) was used. The Cochrane Collaboration tool consists of seven quality domains, and each domain is classified into three levels of risk of bias (low, high, or unclear). The seven domains are: (a) sequence generation, (b) allocation concealment, (c) blinding of participants and personnel, (d) blinding of outcome assessment, (e) incomplete outcome data, (f) selective outcome reporting, and (g) other sources of bias. The quality of the included studies was assessed independently by two out of three reviewers (J.F.R., C.B.K., and M.N.H.).

### Data Synthesis

The effect size was calculated using a standardized mean difference (SMD) [95% confidence interval (CI)] if data were reported as a continuous variable, and different between-study measurement methods were applied. The SMD was translated back to a mean difference for the primary outcome, parent- or clinician rated adaptive functioning using the SD from the control group from the median largest study with the lowest risk of bias.

Adverse effects were expressed as relative risk (RR) with 95% CI, and in the analysis, there were zero adverse effects in both the intervention and control groups, a risk difference meta-analysis (RD; 95% CI) was calculated.

The following subgroups of intervention targets and/or content were applied in the analysis: (a) language, (b) aggression management, (c) training in social skills, and (d) other interventions. For adverse events, the included studies had zero events in both the intervention and control groups; thus, a risk difference meta-analysis (RD; 95% CI) was calculated. A random-effect model was applied for all models. Statistical heterogeneity was quantified using *I*^2^ statistics (23). Since only a few studies were included in each outcome, we did not perform funnel plots to address the potential risk of publication bias.

Review Manager Software (version 5.3) (The Nordic Cochrane Collaboration, Copenhagen, Denmark) (24) was used to perform the analysis and forest plots.

## Results

**Figure 1:**
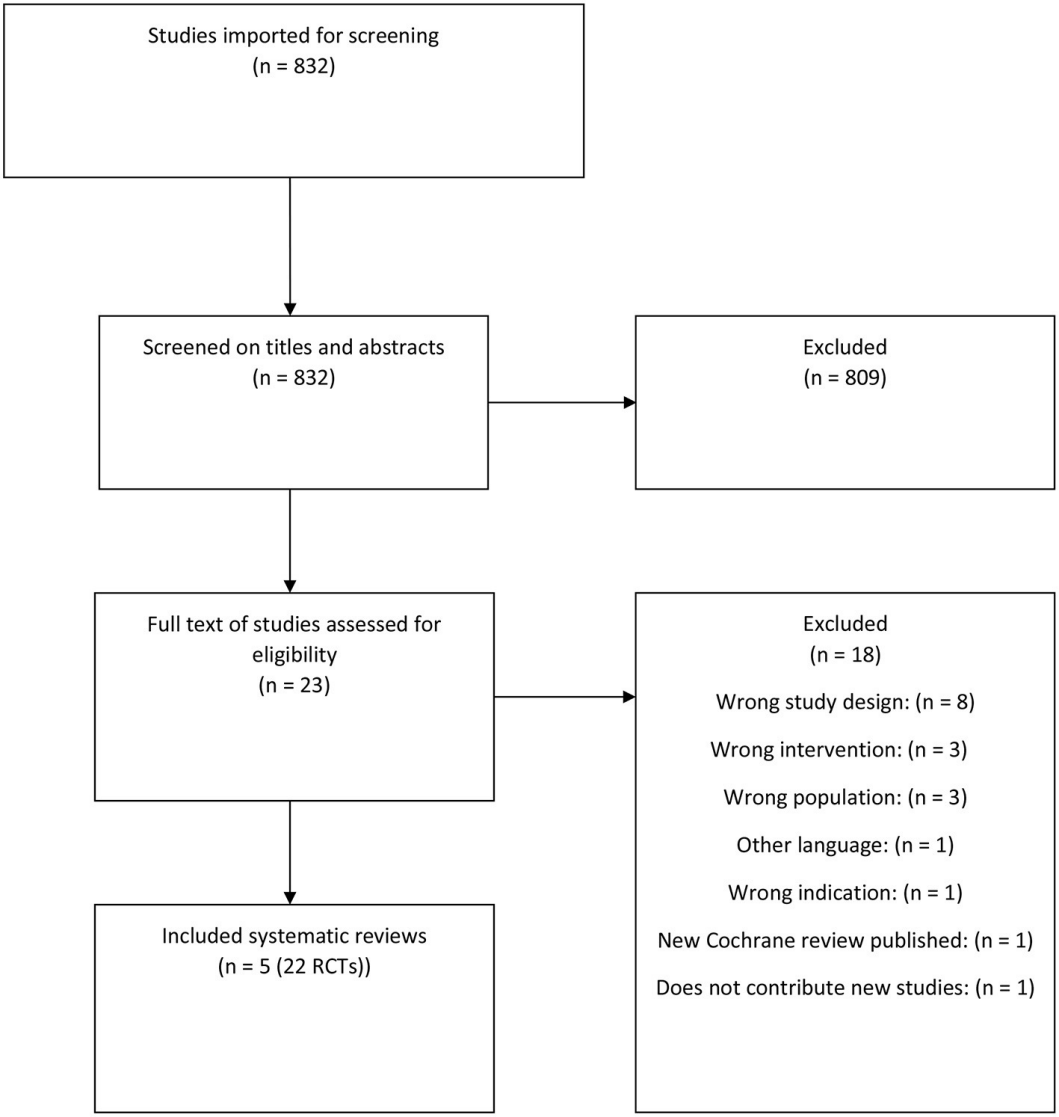
Flowchart of the systematic reviews.

**Figure 2:**
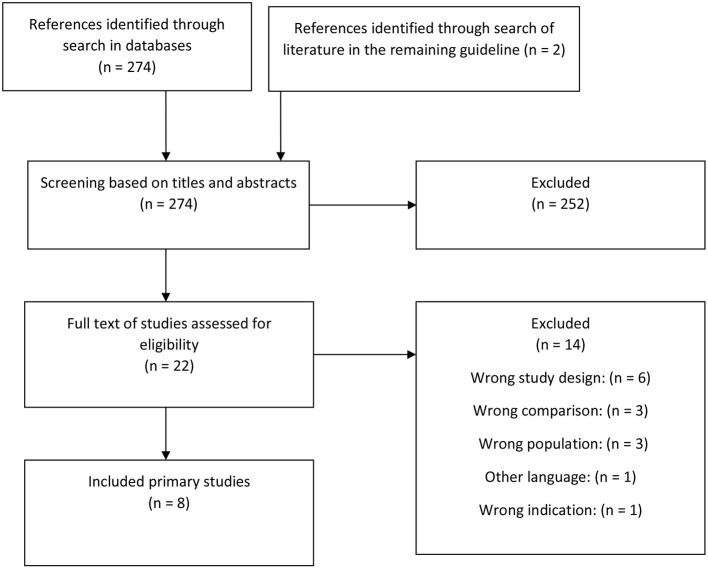
Flowchart of primary studies.

In the initial search, we identified five systematic reviews (10, 13–16) (Figure 1). From these, 22 RCTs were identified (25–46). Through a search for primary literature, an additional six RCTs were identified (47–52) (Figure 2). An additional two studies were included from reference lists in the included articles (53, 54). In total, 30 studies were included in this review and meta-analysis (25–52).

A list of the 14 excluded studies from full-text screening, including reasons for exclusion (55–68), is provided in the Supplementary Table 3.

### Description of the Primary Studies

Characteristics of the included studies are presented in the Supplementary Table 4. The interventions of the included RCTs consisted of PMIs with various target groups and contents. The interventions' commonalities were the parents being direct recipients of the intervention and the parents' intentions to implement and train applied skills with the child. In most studies, the intervention targeted specific areas of ASD (such as joint attention and social-communicative skills). However, in five studies (26, 27, 34, 42, 45), the intervention primarily focused on improving parent-child social interaction and reducing behavioral difficulties and demand-avoidant behavior. In total, parents of 1,934 children and adolescents participated in the studies, and the age range was 16 months to 17 years. Most of the trials were developed for younger children with ASD, with 23 of 30 studies including children 7 years of age and younger, and only seven studies focusing on children below 4 years of age (28–30, 36, 48, 53, 54). No studies investigated adolescents older than 14 years of age, and only four articles included children older than 11 (26, 37, 38, 42). These four interventions either targeted disruptive behavior or supported positive behavior. The intervention period in the included studies were 8 weeks-24 months.

**Figure 3:**
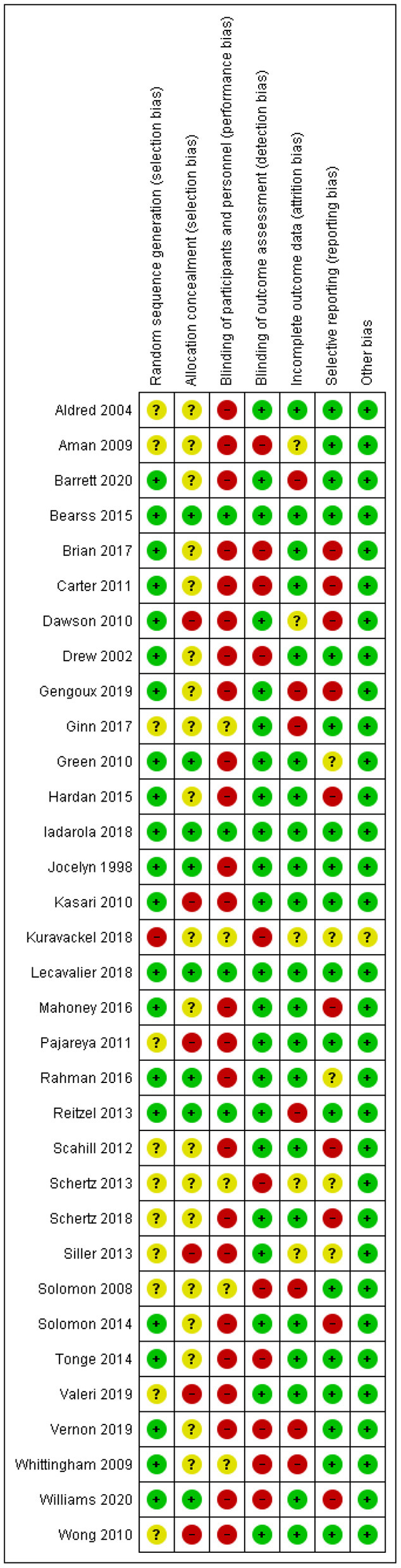
Risk of bias.

The control groups consisted of waitlist or other passive control conditions in 11 of the studies (31, 36, 37, 42, 45–47, 49, 51, 52, 54); treatment/management as usual in 10 of the studies (25, 28, 30, 32, 39, 40, 43, 44, 48, 53); an active control with a less extensive educational program, psycho-education, placebo parent-intervention in eight of the studies; and anti-psychotic medicine (risperidone) alone in a single study (26, 27, 29, 33–35, 38, 41, 50). Four studies (36, 41, 47, 54) either did not report data predefined as primary or secondary outcomes in the present context or did not report data in a manner for them to be included in the meta-analysis. A conclusion as to whether the individual studies were rated with high, low or unclear risk of bias within each domain was reached (Figure 3).

### Synthesis of the Results on Primary Outcome—Adaptive Functioning (Parent- and Clinician-Reported)

**Figure 4:**
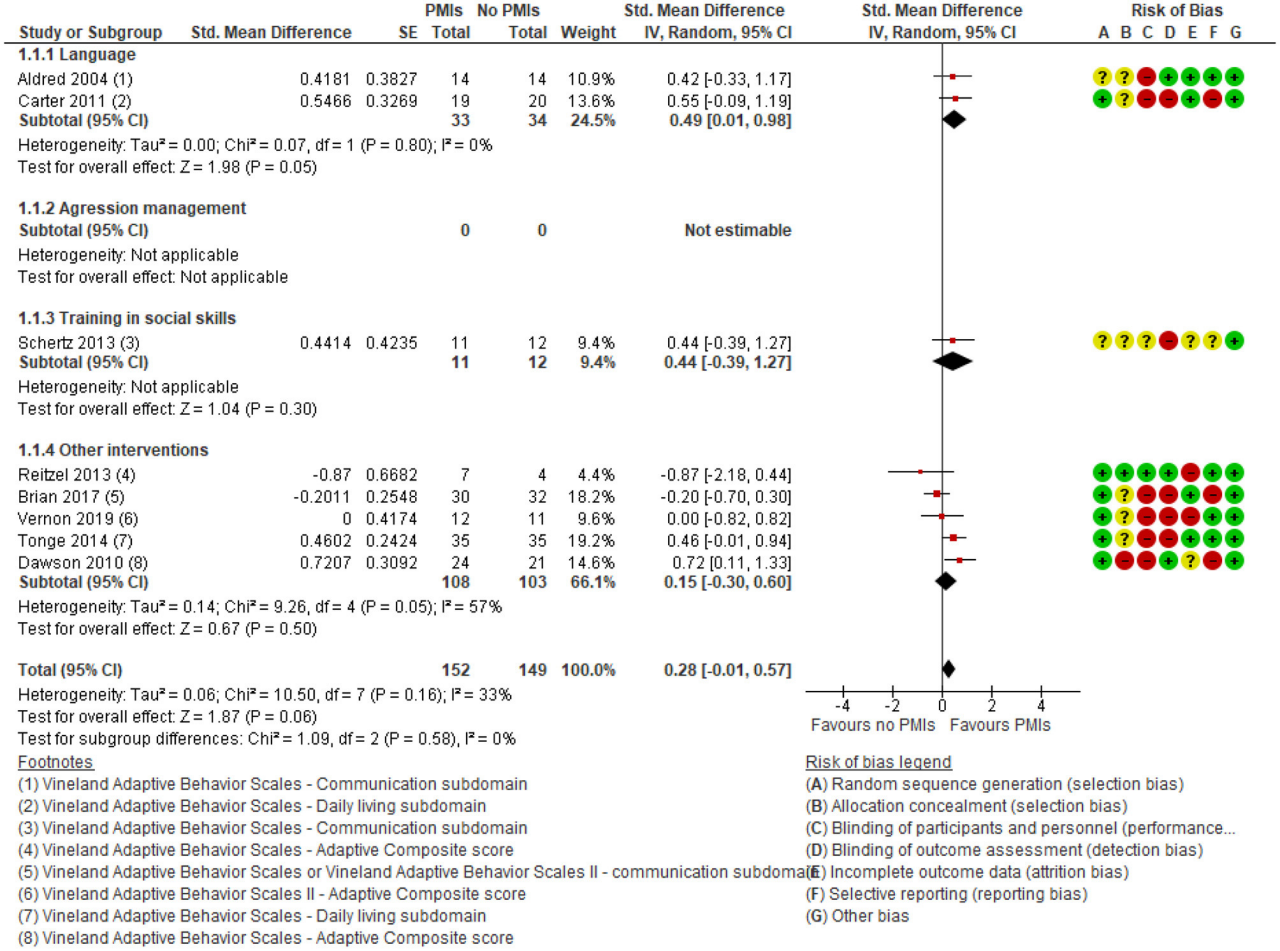
Forest plot of comparison: PMI vs. no PMI, outcome: adaptive functioning, parent-rated, lower is better.

With respect to the primary outcome defined in this review and meta-analysis eight studies reported the effects of PMIs on parent-reported adaptive functioning (25, 28, 29, 40, 44, 48, 52, 53). There was a small but clinically relevant effect [SMD: 0.28 (95% CI:−0.01, 0.57)] (Figure 4), corresponding to an MD on the Vineland scale of 3.5 [95% CI: 0.92, 6.02] calculated from the endpoint SD from the control group in Vernon (52). There was a low degree of heterogeneity (*I*^2^ = 10). All studies included in the meta-analysis used the Vineland Adaptive Behavior Scales 1st or 2nd Edition to assess adaptive functioning; however, they did not all report scores on the same sub-domains. For the meta-analysis, the adaptive composite score was preferred; when not reported, the daily living subdomain was secondarily prioritized. However, three of the included studies (25, 48, 53) did not report any of these scores; for these, the Communications subdomain outcome was used for the meta-analysis. The outcomes were considered parent-rated when based on a parent-rated questionnaire or the clinicians rating a semi-structured interview with parents. One study Tonge et al. (44) combined the parent interview with a simultaneous observation of the child but was categorized as parent-rated since the main information for the scoring was still derived from the parents.

The results showed no subgroup differences in parent-rated adaptive functioning between the different targets of parent-mediated interventions (*p* = 0.58).

For the primary outcome parent-rated adaptive functioning there was low certainty in the effect estimates due to rating down for serious risk of bias due to lack of blinding of participants and outcome assessors, as well as serious risk of imprecision due to few participating children. In conclusion, PMIs may slightly improve parent-rated adaptive functioning.

**Figure 5:**
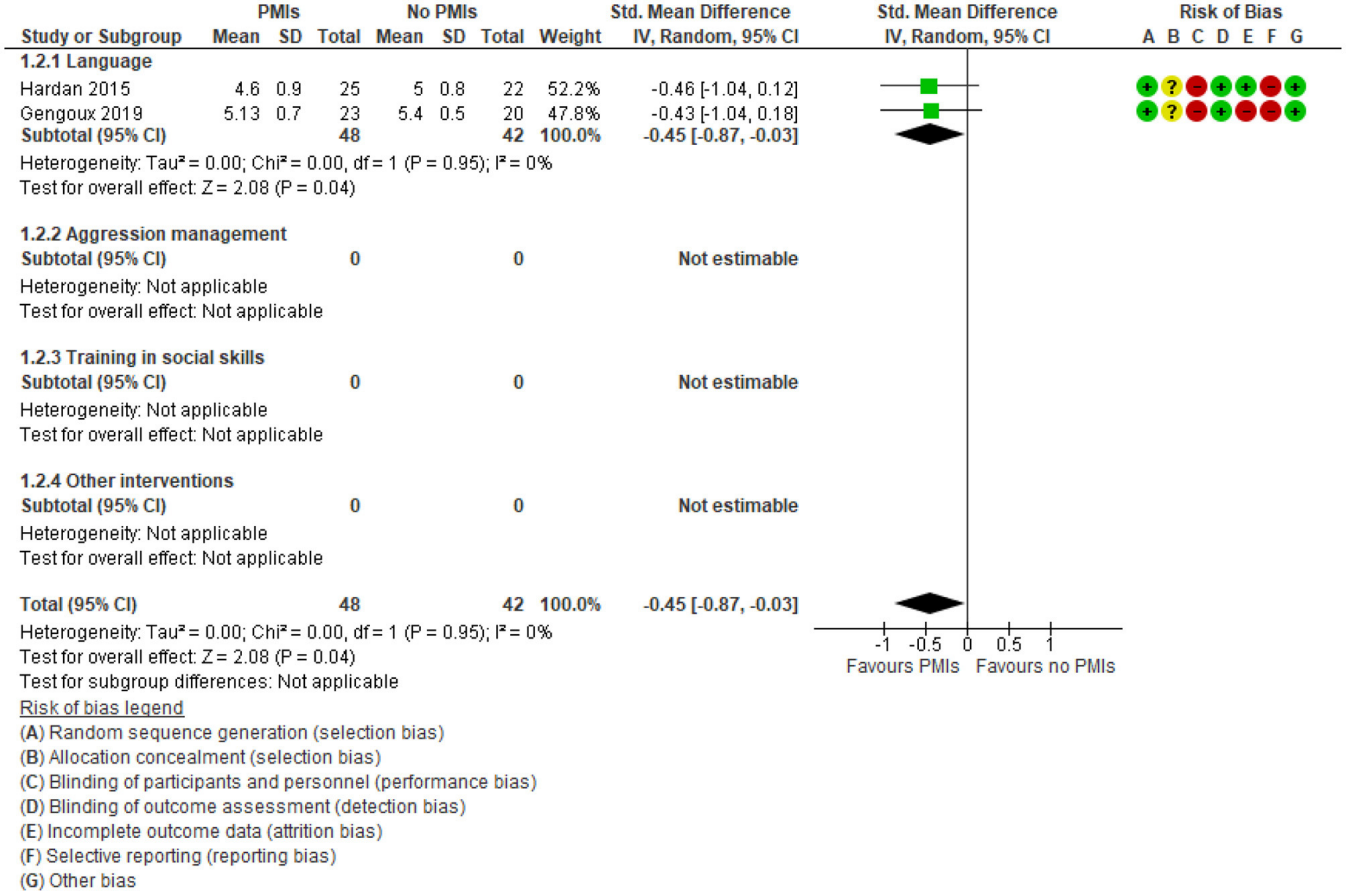
Forest plot of comparison: PMI vs. no PMI, outcome: adaptive functioning, clinician-rated, (CGI severity), lower is better.

Only 2 studies of PMIs focusing on language reported clinician-rated adaptive functioning (33, 49), and the studies found no effect of PMIs [SMD −0.45 (95% CI: −0.87, −0.03) (Figure 5) corresponding to a MD on CGI severity scale: −0.36 (95% CI: −0.70, −0.02)]. The degree of heterogeneity was low (*I*^2^ = 0%). There was very low certainty in the evidence on clinician-rated adaptive functioning due to rating down for serious risk of bias due to problems with lack of blinding of participants and outcome assessors and very serious risk of imprecision due to few participating children and wide confidence intervals. Thus, it is uncertain if the PMIs increase the clinician-rated adaptive functioning.

### Synthesis of the Results on Secondary Outcomes

**Figure 6:**
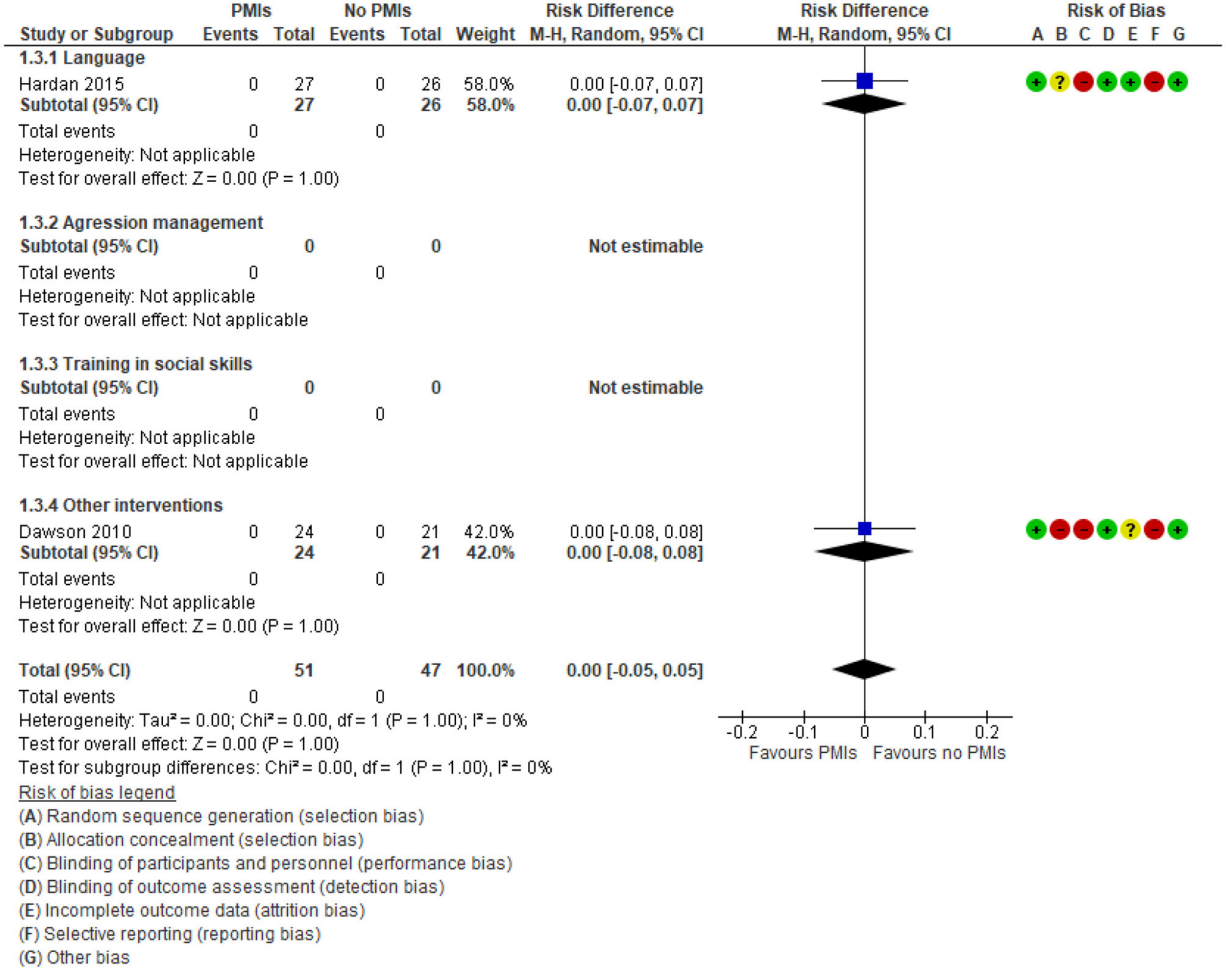
Forest plot of comparison: PMI vs. no PMI, outcome: adverse effects.

A single study (44) reported on parent-rated adaptive functioning after 6 months of follow-up on Vineland Adaptive Behavior Scales Daily Living subscale. This study did not report an end of treatment effect, thus was included in the meta-analysis of the primary outcome (Figure 4). The study showed significant improvement in the daily living subdomain of VABS [effect size −0.62 (95% CI: −1.10, −0.13)] and the socialization subdomain [effect size−0.60 (95% CI: −1.09, −0.12)] in the PMI group compared to a psychoeducative group (44). Adverse effects were only reported in 2 of 30 studies (29, 33) and reported no adverse effects in any of the two groups (Figure 6). One of the studies compared the Early Start Denver Model to treatment as usual (29), and the other compared Pivotal Response Training to 12 sessions of psychoeducation (33). There was low certainty in the evidence due to rating down for very serious risk of imprecision because of few participating children and few studies reporting adverse effects. Thus, PMIs may not cause substantial adverse effects.

**Figure 7:**
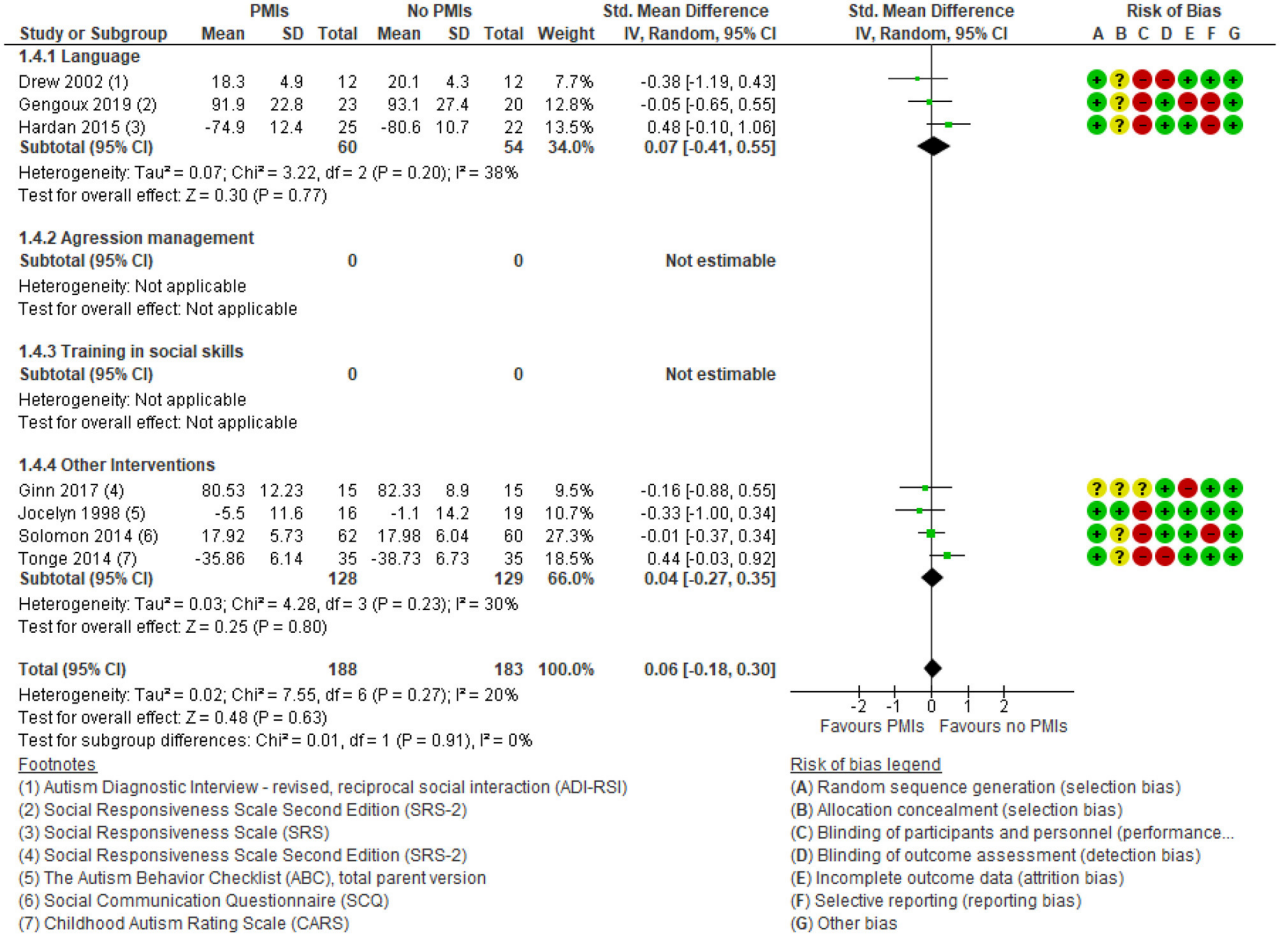
Forest plot of comparison: PMI vs. no PMI, outcome: autism core symptoms, parent-rated.

Seven studies reported the effect of PMIs on parent-rated core symptoms of ASD (30, 31, 33, 35, 43, 44, 49). No relevant effect was found [SMD: 0.06 (95% CI: −0.18, 0.30)] (Figure 7). The degree of heterogeneity was low for the parent-rated outcome (*I*^2^ = 20%). The results showed no subgroup differences between the different targets of PMIs, e.g., language and disruptive behavior (*p* = 0.91). There was low certainty in the evidence due to rating down for serious risk of bias because of lack of blinding of participants and outcome assessors, as well as serious risk of imprecision due to few participating children. Thus, PMIs may result in little or no clinical relevant change in parent-rated autism core symptoms.

**Figure 8:**
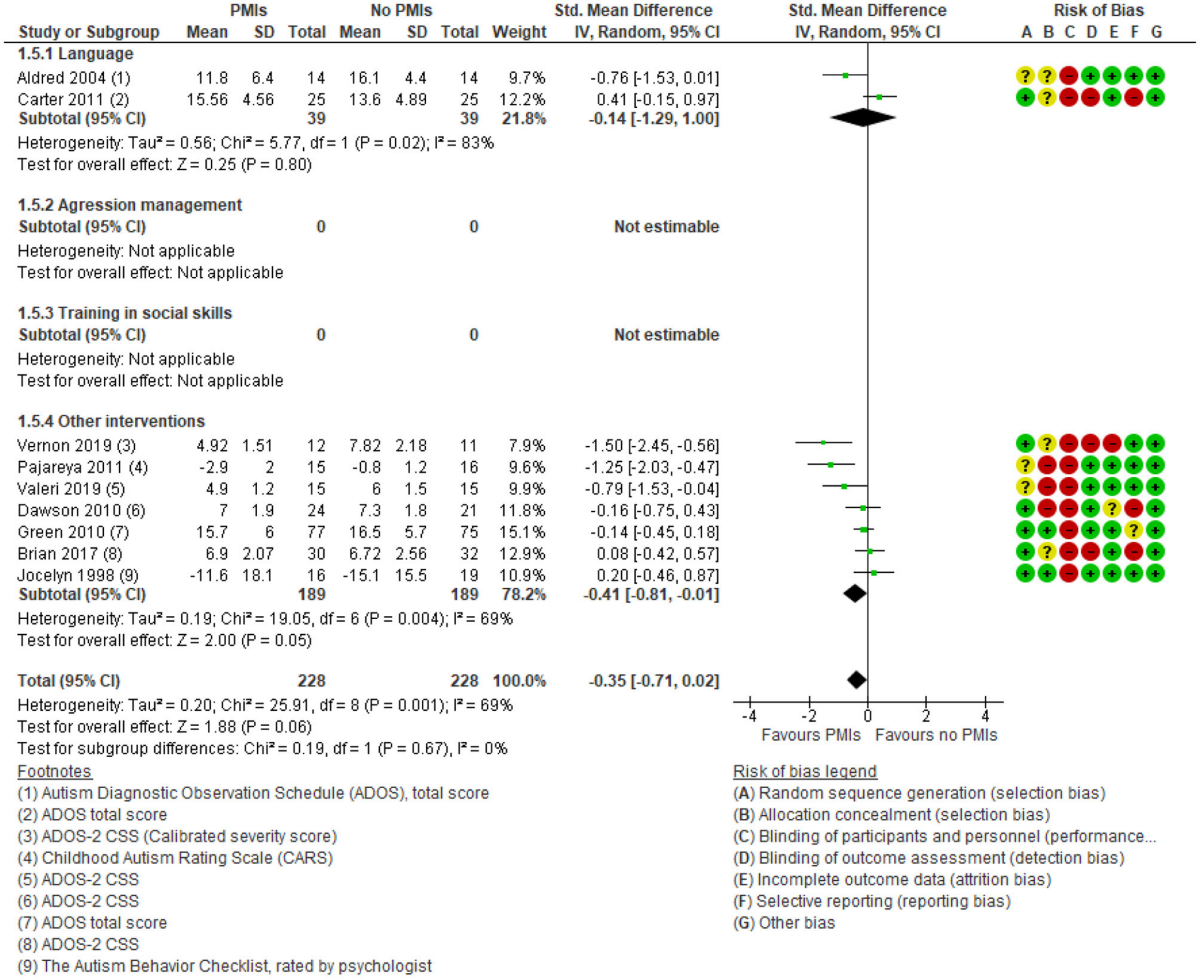
Forest plot of comparison: PMI vs. no PMI, outcome: autism core symptoms, clinician-rated.

Nine studies reported a small effect of PMIs on clinician-rated core symptoms of ASD [SMD: −0.35 (95% CI: −0.71, 0.02)] (Figure 8) (25, 28, 29, 32, 35, 39, 48, 50, 52). The degree of heterogeneity was considerable for the clinician-rated outcome (*I*^2^ = 69%). The results showed no subgroup differences between the different targets of PMIs, e.g., language and disruptive behavior (*p* = 0.67). There was low certainty in the evidence due to rating down for serious risk of bias because of lack of blinding of participants and outcome assessors, as well as serious risk of imprecision due to few participating children. Thus, PMIs may slightly improve clinician-rated autism core symptoms.

**Figure 9:**
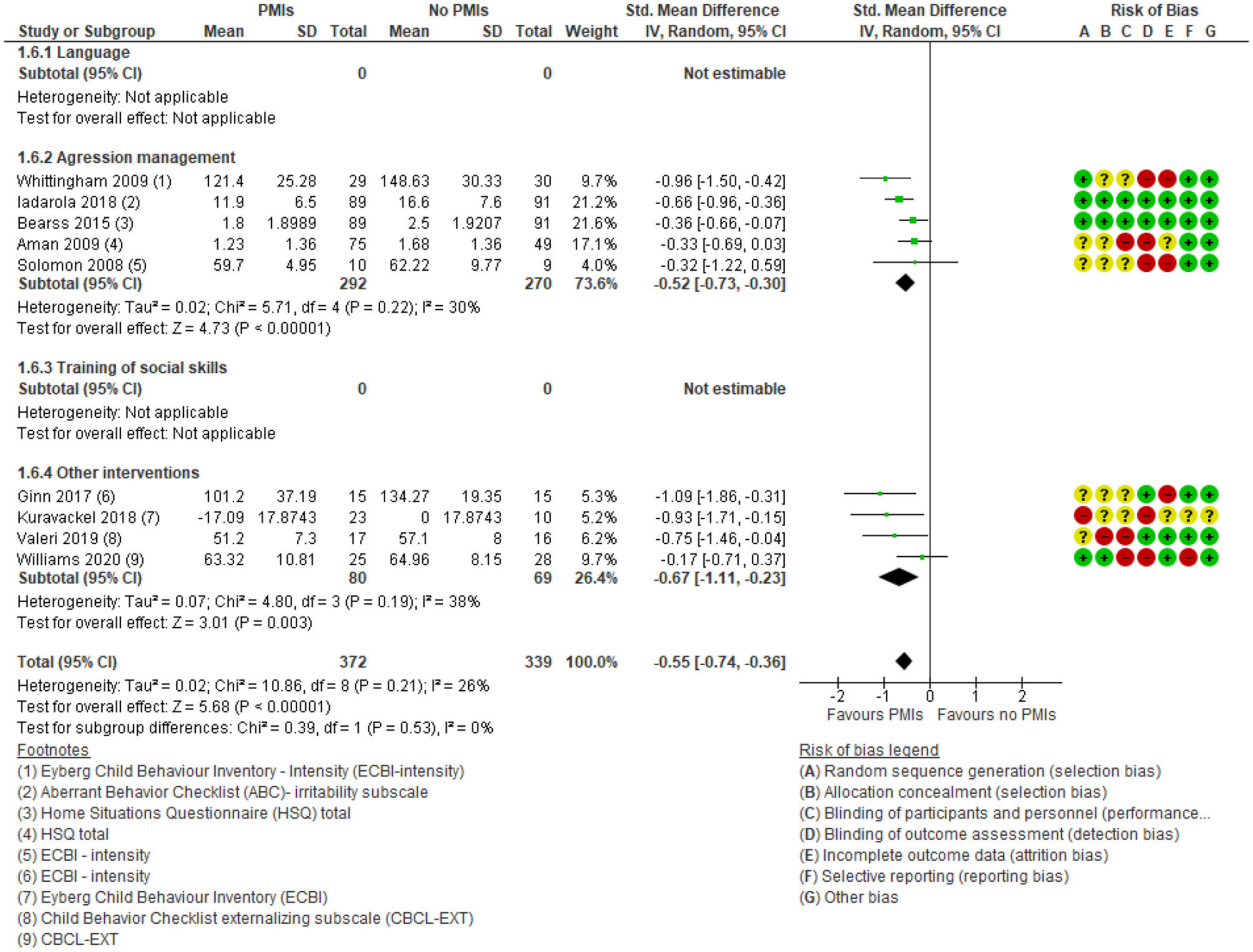
Forest plot of comparison: PMI vs. no PMI, outcome: disruptive behavior, parent-rated.

Regarding parent-rated disruptive behavior, there was a moderate and clinically relevant effect of PMIs [SMD: −0.55 (95% CI: −0.74, −0.36)] (Figure 9). None of the language interventions reported on this outcome. There was a low degree of heterogeneity (*I*^2^ = 26%), and the results showed no subgroup differences between the different targets of PMIs (*p* = 0.53). There was moderate certainty in the evidence due to rating down for serious risk of bias because of lack of blinding of participants and outcome assessors. Thus, PMIs probably improves parent-rated disruptive behavior considerably.

**Figure 10:**
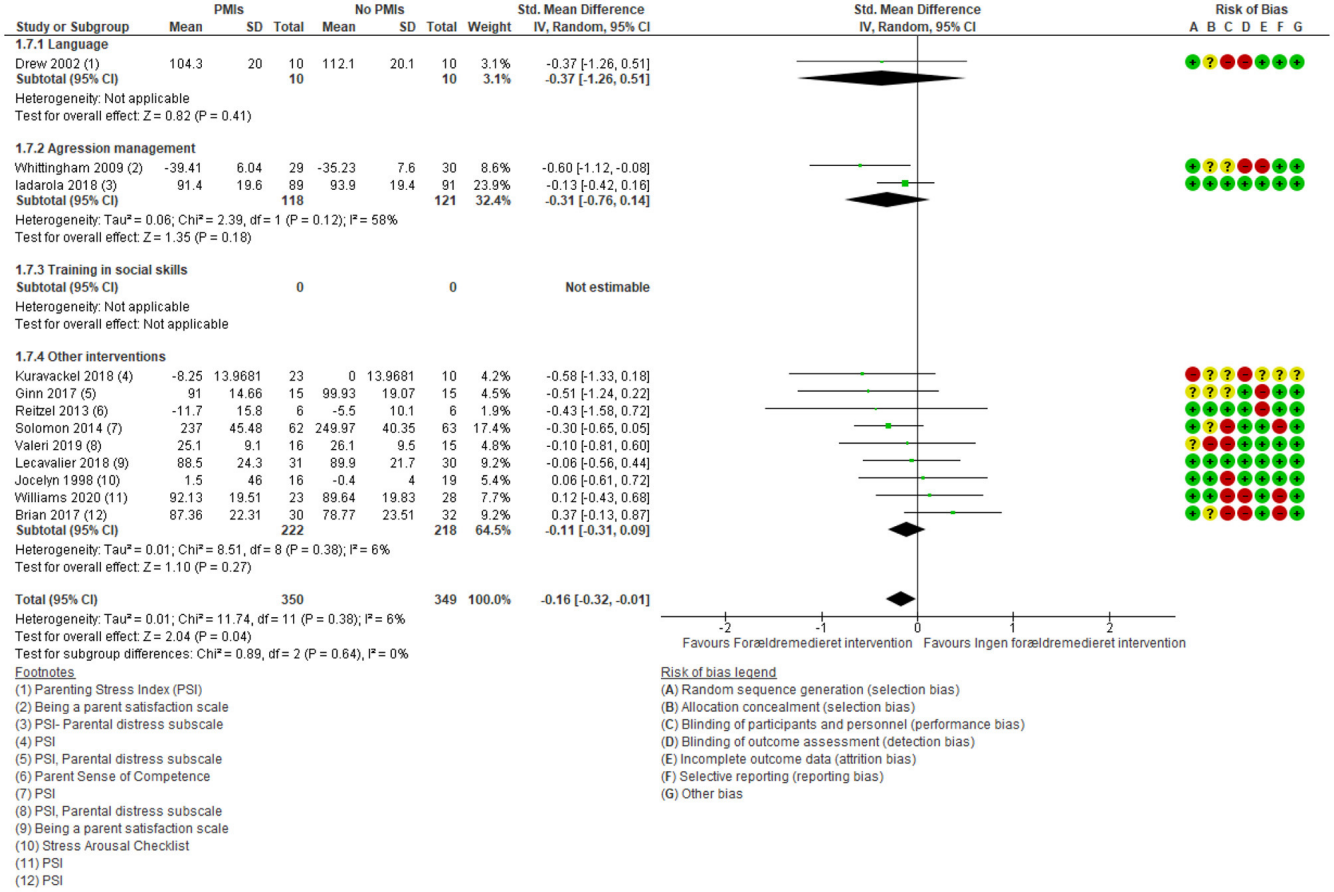
Forest plot of comparison: PMI vs. no PMI intervention, outcome: parental well-being.

Parental well-being showed a small and not clinically relevant effect of PMIs [SMD: −0.16 (95% CI: −0.32, −0.01)] (Figure 10). There was a low degree of heterogeneity (*I*^2^ = 6%), and the results showed no subgroup differences between the different targets of PMIs (*p* = 0.64). There was low certainty in the evidence due to rating down for very serious risk of imprecision because of few participating children and wide confidence intervals (Supplementary Table 5). Thus, PMIs may result in little or no clinically relevant change in parental well-being.

None of the included studies reported on child anxiety or parent-rated quality of life of the child.

## Discussion

The current systematic review identified 30 RCTs of PMIs for children and adolescents with ASD. The results showed a clinically relevant effect on parent-rated adaptive functioning but no effect when the outcome was rated by a clinician. Moreover, PMIs may be a valuable treatment for disruptive behavior in children and adolescents with ASD. There was no effect on core symptoms of ASD (both parent- and clinician-rated), parent-rated adaptive functioning after 6 months or adverse effects. No studies reported on anxiety or parent-rated quality of life of the child. The certainty of the effect estimates reported in the studies was moderate to very low due to serious risk of bias and very serious risk of imprecision; thus, the effect of PMIs on children and adolescents with ASD is still uncertain. A qualitative synthesis of the studies revealed significant variability across studies.

Meanwhile, previous reviews (10, 13–16) presented evidence in favor of PMIs and emphasized the positive effect of PMIs on joint attention and social interaction in the parent-child dyad, which was not among the prespecified outcomes of this review (10, 15, 16).

This meta-analysis suggests a clinically relevant effect of PMIs for children and adolescents with ASD on parent-rated adaptive functioning, which was not found in the review and meta-analysis by Oono et al. (10). This may be explained by the inclusion of more studies in our review compared to Oono et al. (10) and thus an increase in power. This is a strong indicator of the efficacy of PMIs, since none of the interventions directly affects adaptive functioning, and the positive outcome on adaptive functioning could be a cascading effect of the PMIs.

The results from the meta-analysis align with results from previous reviews regarding favorable results of child disruptive behavior (14, 15). Interestingly, interventions targeting behavior as well as social communication and joint attention for younger children demonstrated positive effects of PMIs on disruptive behavior (50).

Previous reviews have also identified minor improvements in core symptoms of ASD in children participating in PMIs (10, 13), which was not confirmed in this meta-analysis. However, the clinician-rated core symptom measures favored of the PMIs, with a non-significant but clinically relevant effect.

As early interventions have been developed for children with ASD over the past several years (69), it is expected that most studies target younger children when searching for PMIs. This was confirmed in the current review, where only two of 30 included studies targeted children 5 years of age and older (38, 42), although the age range of the study population in this review 18 months to 17 years. The age range was suggested by the scientific committee, and interest associations which were both appointed by the Danish Health Authority to develop national Clinical Practice Guidelines in Denmark. The inclusion of both children and adolescents was relevant for most of the frameworks in the guideline development and was therefore applied to all of the PICOs.

Typically, interventions targeting social communication are recommended to younger children. This underlines the necessity of using interventions appropriate for the child's age and developmental stage. With respect to the remaining secondary outcomes, a clinically relevant effect regarding clinician rated core symptoms of ASD was seen in favor of PMIs. Moreover, the interventions targeting conduct problems were provided to younger children with ASD aged 2–9 years of age (27, 34, 45); interestingly, some of the studies extended the intervention to be applied to children 4 and 5 years of age to 12 and 13-year-old children (26, 42), suggesting that these kinds of interventions are effective at improving positive behavior in both younger and older children.

### Strengths and Limitations of the Included Studies

A critical limitation of the included studies is that different outcome measures were used to assess the same effect; and when they used the same assessment tools, they were administered differently. For example, the Vineland Adaptive Behavior Scale (VABS) was used to assess adaptive functioning, and it was administered either as an interview or a parent-reported questionnaire (70). Another limitation is a generally small sample size in several studies.

### Strengths and Limitations of the Methodology of the Present Systematic Review and Meta-Analysis

A major strength of this systematic review and meta-analysis is that it was performed according to principles described in GRADE and PRISMA as well as the PICO framework. However, from a resource saving perspective, we chose a stepwise literature search by initially searching for existing reviews to identify eligible studies, followed by a search for primary studies based on the latest search date of an existing high-quality systematic review, and finally screening reference lists of included studies and conferring content experts (working group at the Danish Health Authority). *Post-hoc*, we identified three articles with a total of 307 participants (71–73) (Supplementary Table 6) that were not identified in any of the included reviews by consulting content experts or in the reference list of the included studies. Since the ability to identify relevant studies is mainly dependent on the scope and search quality of the existing reviews, we acknowledge that our search may have been limited regarding both search specificity (recall) and sensitivity (precision). However, the results did not change substantially when performing *post-hoc* sensitivity analysis and including results from the unidentified studies. However, the increased power strengthened the positive results of PMIs on both parent-rated adaptive functioning [SMD: 0.27 (95% CI: 0.02, 0.52)] and clinician-rated autism core symptoms [SMD: −0.34 (95% CI: −0.64, −0.03)] (Supplementary Table 7).

### Future Research

To estimate the effect of PMIs, further research with large high-quality RCTs investigating manualized interventions and following standardized principles for trial design, content and reporting is needed. Interventions previously investigated in RCTs need replication studies to build on the evidence of the intervention. In future research, it could be interesting to investigate the association between contextual factors, such as age and effect size, to address which children are most likely to benefit from PMIs. A need for measurement consistency still remains and is recommended in future research, to improve comparison between studies. Furthermore, there is a need to assess a core outcome set to investigate the importance of anxiety, parent-rated adaptive functioning after a minimum of 6 months and parent-rated child's quality of life.

## Conclusion

When PMIs are delivered to children with ASD, it is recommended to use manualized interventions targeting autism spectrum disorders, and the characteristics of the included PMIs are that the child should be directly or indirectly involved in the intervention. However, the parents are the primary participants in the treatment and must actively train the different skills included in the intervention, both during the sessions and/or at home between sessions. The intervention must be adapted to the age and development of the child.

Based on the current evidence, there appears to be a benefit of providing PMIs to parents of children and adolescents with ASD concerning adaptive functioning and disruptive behavior reported by the parents. Perhaps by enhancing the parents' understanding and management of their child's pervasive disorder, it seems that the parents are empowered, which is supported by the trend toward improving parental well-being. As expected, there were minor differences between the intervention and control groups in changing core symptoms of ASD. PMIs may slightly improve clinician-rated autism core symptoms. Even though there were few reports on adverse effects, any adverse effects of the PMIs were considered insignificant and few. Adverse effects should be included in future studies. However, since the evidence base's certainty is low, the limitations of the current literature hinder the possibility of drawing any solid conclusions, and more well-designed, high-quality clinical trials of sufficient duration are required.

## Author Contributions

MR, JR, BP, CK, ST, and MH: methodology. MR, JR, BP, CK, and MH: data curation. MH: formal analysis, visualization, supervision, and project administration. All authors investigation, resources, writing—original draft preparation, writing—review and editing, funding acquisition, and read and agreed to the published version of the manuscript.

## Funding

This research was funded by the Danish Health Authority. The Parker Institute was supported by a core grant by the OAK foundation (OCAY-18-774-OFIL).

## Conflict of Interest

The authors declare that the research was conducted in the absence of any commercial or financial relationships that could be construed as a potential conflict of interest.

## Publisher's Note

All claims expressed in this article are solely those of the authors and do not necessarily represent those of their affiliated organizations, or those of the publisher, the editors and the reviewers. Any product that may be evaluated in this article, or claim that may be made by its manufacturer, is not guaranteed or endorsed by the publisher.

## Supplementary Material

Supplementary Table 1 PRISMA checklist.

Supplementary Table 2 Search description.

Supplementary Table 3 Characteristics of excluded studies.

Supplementary Table 4 Characteristics of the included studies.

Supplementary Table 5 Summary of findings table.

Supplementary Table 6 Characteristics of *post-hoc* identified studies.

Supplementary Table 7 *post-hoc* analyses.
