# Supplementary material for: Parent-Mediated Interventions for Children and Adolescents With Autism Spectrum Disorders: A Systematic Review and Meta-Analysis
Source: Front Psychiatry. 2021 Nov 12;12:773604. doi: 10.3389/fpsyt.2021.773604 (PMC8632873; doi:10.3389/fpsyt.2021.773604)
Supplement: Supplementary Table 2 — Search description. [file Table_2.DOCX]

**Table S2. Search description**

**Search protocol - National Clinical Guidelines**

| Project title / aspect | National Clinical Guidelines for treatment of autism spectrum disorder in children and adolescents - Secondary literature |
| --- | --- |
| Expert consultantProject manager **Method consultant** | Marie Louise Rimestad Marie Herlev Ahrenfeldt, SST  Mina Händel, SST |
| Search specialist | Birgitte Holm Petersen, SST |

| **Background** | PICO question: Should parent-mediated interventions be offered to parents of children aged 18 months – 17 years with autism? |
| --- | --- |
| **Search terms** | Defined in the article |
| **Inclusion and exclusion criteria** | Language: English, Danish, Norwegian and Swedish  Population: children and adolescents  Publication types: Randomized controlled trials / RCT |

**Information Sources**

| **DATABASE** | **INTERFACE** | **Search date** |
| --- | --- | --- |
| **Medline incl. Cochrane** | OVID | March 17, 2020 |
| **EMBASE incl. Cochrane** | OVID | March 16, 2020 – March 19, 2020 |
| **PSYCINFO** | OVID | March 16, - March 19, 2020 |
| **CINAHL** | EBSCO | March 19, 2020 |
| **ERIC** | EBSCO | March 19, 2020 |

**Note**

- Search terms and inclusion and exclusion criteria are adapted each database.
- Doublets are removed, when possible, by RefWorks. The found references are transferred to Covidence
- Full-text articles are presented in Covidence in pdf format
- Search strategies for every single database are reported

**Search strategy:**

**PICO 1 prim. (parent)**

**Medline (190320)**

Database(s): **Ovid MEDLINE(R) and Epub Ahead of Print, In-Process & Other Non-Indexed Citations, Daily and Versions(R)**1946 to March 17, 2020

Search Strategy:

| **#** | **Searches** | **Results** |
| --- | --- | --- |
| 1 | (autism* or ASD).mp. [mp=title, abstract, original title, name of substance word, subject heading word, floating sub-heading word, keyword heading word, organism supplementary concept word, protocol supplementary concept word, rare disease supplementary concept word, unique identifier, synonyms] | 50383 |
| 2 | Autism Spectrum Disorder*.mp. or Autism Spectrum Disorder/ | 24147 |
| 3 | Autism Disorder*.mp. | 123 |
| 4 | Autistic Disorder/ or Autistic Disorder*.mp. | 20598 |
| 5 | autistic*.mp. | 25127 |
| 6 | Asperger Syndrome/ or asperger*.mp. | 2743 |
| 7 | asperger*.mp. | 2743 |
| 8 | Neurodevelopmental Disorders/ or neurodevelopmental disorder*.mp. | 10757 |
| 9 | Child Development Disorders, Pervasive.mp. or Child Development Disorders, Pervasive/ | 6532 |
| 10 | or/1-9 | 64805 |
| 11 | parent intervention*.mp. | 159 |
| 12 | parent mediated intervention*.mp. | 69 |
| 13 | parenting intervention*.mp. | 814 |
| 14 | (parent support* or parents support).mp. [mp=title, abstract, original title, name of substance word, subject heading word, floating sub-heading word, keyword heading word, organism supplementary concept word, protocol supplementary concept word, rare disease supplementary concept word, unique identifier, synonyms] | 691 |
| 15 | Parents/ or Parenting/ or parent support program*.mp. | 74848 |
| 16 | ((Parent* or parenting*) and (exercise* or training*)).mp. [mp=title, abstract, original title, name of substance word, subject heading word, floating sub-heading word, keyword heading word, organism supplementary concept word, protocol supplementary concept word, rare disease supplementary concept word, unique identifier, synonyms] | 16190 |
| 17 | parent training*.mp. | 1286 |
| 18 | peer-mediated intervention*.mp. | 50 |
| 19 | (peer-mediated intervention* or PMI).mp. [mp=title, abstract, original title, name of substance word, subject heading word, floating sub-heading word, keyword heading word, organism supplementary concept word, protocol supplementary concept word, rare disease supplementary concept word, unique identifier, synonyms] | 2551 |
| 20 | Parents/ or Parenting/ or parent support program*.mp. | 74848 |
| 21 | Psychosocial Support Systems/ or psychosocial support system*.mp. | 498 |
| 22 | or/11-21 | 90035 |
| 23 | 10 and 22 | 3049 |
| 24 | (((random* or cluster-random* or control?ed or crossover or cross-over or blind* or mask*) adj4 (trial*1 or study or studies or analy*)) or rct).ti,ab,kw,kf. | 685019 |
| 25 | (placebo* or single-blind* or double-blind* or triple-blind*).ti,ab. | 278877 |
| 26 | ((single or double or triple) adj1 (blind* or mask*)).ti,ab. | 170697 |
| 27 | 24 or 25 or 26 | 772734 |
| 28 | 23 and 27 | 285 |
| 29 | limit 28 to yr="2017 - 2020" | 118 |

**Embase (190320)**

Database(s): **Embase**1996 to 2020 Week 11

Search Strategy:

| **#** | **Searches** | **Results** |
| --- | --- | --- |
| 1 | (autism* or ASD).mp. [mp=title, abstract, heading word, drug trade name, original title, device manufacturer, drug manufacturer, device trade name, keyword, floating subheading word, candidate term word] | 77523 |
| 2 | Autism Spectrum Disorder*.mp. or Autism Spectrum Disorder/ | 52282 |
| 3 | Autism Disorder*.mp. | 192 |
| 4 | Autistic Disorder/ or Autistic Disorder*.mp. | 21872 |
| 5 | autistic*.mp. | 14067 |
| 6 | Asperger Syndrome/ or asperger*.mp. | 5276 |
| 7 | asperger*.mp. | 5276 |
| 8 | Neurodevelopmental Disorders/ or neurodevelopmental disorder*.mp. | 144712 |
| 9 | Child Development Disorders, Pervasive.mp. or Child Development Disorders, Pervasive/ | 18832 |
| 10 | or/1-9 | 215628 |
| 11 | parent intervention*.mp. | 188 |
| 12 | parent mediated intervention*.mp. | 87 |
| 13 | parenting intervention*.mp. | 864 |
| 14 | (parent support* or parents support).mp. [mp=title, abstract, heading word, drug trade name, original title, device manufacturer, drug manufacturer, device trade name, keyword, floating subheading word, candidate term word] | 849 |
| 15 | Parents/ or Parenting/ or parent support program*.mp. | 95889 |
| 16 | ((Parent* or parenting*) and (exercise* or training*)).mp. [mp=title, abstract, heading word, drug trade name, original title, device manufacturer, drug manufacturer, device trade name, keyword, floating subheading word, candidate term word] | 20834 |
| 17 | parent training*.mp. | 1491 |
| 18 | peer-mediated intervention*.mp. | 54 |
| 19 | (peer-mediated intervention* or PMI).mp. [mp=title, abstract, heading word, drug trade name, original title, device manufacturer, drug manufacturer, device trade name, keyword, floating subheading word, candidate term word] | 3461 |
| 20 | Parents/ or Parenting/ or parent support program*.mp. | 95889 |
| 21 | Psychosocial Support Systems/ or psychosocial support system*.mp. | 17504 |
| 22 | or/11-21 | 131385 |
| 23 | 10 and 22 | 9040 |
| 24 | (((random* or cluster-random* or control?ed or crossover or cross-over or blind* or mask*) adj4 (trial*1 or study or studies or analy*)) or rct).ti,ab,kw. | 857109 |
| 25 | (placebo* or single-blind* or double-blind* or triple-blind*).ti,ab. | 321893 |
| 26 | ((single or double or triple) adj1 (blind* or mask*)).ti,ab. | 186130 |
| 27 | 24 or 25 or 26 | 953110 |
| 28 | 23 and 27 | 693 |
| 29 | limit 28 to yr="2017 - 2020" | 198 |

**Psycinfo (190320)**

Database(s): **APA PsycInfo**1806 to March Week 3 2020

Search Strategy:

| **#** | **Searches** | **Results** |
| --- | --- | --- |
| 1 | (autism* or ASD).mp. [mp=title, abstract, heading word, table of contents, key concepts, original title, tests & measures, mesh] | 53193 |
| 2 | Autism Spectrum Disorder*.mp. or Autism Spectrum Disorder/ | 45854 |
| 3 | Autism Disorder*.mp. | 102 |
| 4 | Autistic Disorder/ or Autistic Disorder*.mp. | 11652 |
| 5 | autistic*.mp. | 21740 |
| 6 | Asperger Syndrome/ or asperger*.mp. | 4467 |
| 7 | asperger*.mp. | 4467 |
| 8 | Neurodevelopmental Disorders/ or neurodevelopmental disorder*.mp. | 6220 |
| 9 | Child Development Disorders, Pervasive.mp. or Child Development Disorders, Pervasive/ | 4447 |
| 10 | or/1-9 | 59883 |
| 11 | parent intervention*.mp. | 296 |
| 12 | parent mediated intervention*.mp. | 93 |
| 13 | parenting intervention*.mp. | 1175 |
| 14 | (parent support* or parents support).mp. [mp=title, abstract, heading word, table of contents, key concepts, original title, tests & measures, mesh] | 1322 |
| 15 | Parents/ or Parenting/ or parent support program*.mp. | 48030 |
| 16 | ((Parent* or parenting*) and (exercise* or training*)).mp. [mp=title, abstract, heading word, table of contents, key concepts, original title, tests & measures, mesh] | 24578 |
| 17 | parent training*.mp. | 8256 |
| 18 | peer-mediated intervention*.mp. | 193 |
| 19 | (peer-mediated intervention* or PMI).mp. [mp=title, abstract, heading word, table of contents, key concepts, original title, tests & measures, mesh] | 482 |
| 20 | Parents/ or Parenting/ or parent support program*.mp. | 48030 |
| 21 | Psychosocial Support Systems/ or psychosocial support system*.mp. | 68 |
| 22 | exp family Interbvention/ or family intervention*.mp. [mp=title, abstract, heading word, table of contents, key concepts, original title, tests & measures, mesh] | 5022 |
| 23 | or/11-22 | 74052 |
| 24 | 10 and 23 | 3900 |
| 25 | (((random* or cluster-random* or control?ed or crossover or cross-over or blind* or mask*) adj3 (trial*1 or study or studies or analy*)) or rct).ti,ab,id. | 97572 |
| 26 | ((placebo* or single-blind* or double-blind* or triple-blind* or (single or double or triple)) adj2 (blind* or mask*)).ti,ab,id. | 26060 |
| 27 | 25 or 26 | 104593 |
| 28 | 24 and 27 | 259 |
| 29 | limit 28 to yr="2017 - 2020" | 90 |

**Cinahl (190320)**

| Thursday, March 19, 2020 8:03:48 AM |
| --- |

| **#** | **Query** | **Results** |  |  |  |
| --- | --- | --- | --- | --- | --- |
| S16 | S9 AND S10 AND S14  Limiters - Published Date: 20170101-20201231 | 31 |  |  |  |
| S15 | S9 AND S10 AND S14 | 104 |  |  |  |
| S14 | S11 OR S12 OR S13 | 535,960 |  |  |  |
| S13 | (MH "Randomized Controlled Trials+") OR "Randomized Controlled Trial" | 139,413 |  |  |  |
| S12 | (placebo* or single-blind* or double-blind* or triple-blind* or ((single or double or triple) N1 (blind* or mask*)) | 114,369 |  |  |  |
| S11 | (((random* or cluster-random* or control#ed or crossover or cross-over or blind* or mask*) N3 (trial* or study or studies or analy*)) or rct) | 525,131 |  |  |  |
| S10 | S1 OR S2 OR S3 OR S4 OR S5 OR S6 | 4,365 |  |  |  |
| S9 | S7 OR S8 | 44,023 |  |  |  |
| S8 | (MH "Autistic Disorder") OR (MH "Developmental Disabilities") OR (MH "Asperger Syndrome") OR "autism" | 42,268 |  |  |  |
| S7 | autism* or ASD | 30,773 |  |  |  |
| S6 | peer mediated intervention* | 51 |  |  |  |
| S5 | parent support* | 694 |  |  |  |
| S4 | parenting intervention* | 739 |  |  |  |
| S3 | parent mediated intervention* | 56 |  |  |  |
| S2 | parent intervention* | 207 |  |  |  |
| S1 | parent Training* | 2,911 |  |  |  |

**ERIC (190320)**

| Thursday, March 19, 2020 7:51:21 AM |
| --- |

| **#** | **Query** | **Results** |  |  |  |
| --- | --- | --- | --- | --- | --- |
| S20 | S15 AND S18  Limiters - Date Published: 20170101-20191231 | 17 |  |  |  |
| S19 | S15 AND S18 | 52 |  |  |  |
| S18 | S16 OR S17 | 3,008 |  |  |  |
| S17 | DE "Randomized Controlled Trials" | 1,512 |  |  |  |
| S16 | Randomized Controlled Trial* or RCT | 3,008 |  |  |  |
| S15 | S3 AND S14 | 1,833 |  |  |  |
| S14 | S4 OR S5 OR S6 OR S7 OR S8 OR S9 OR S10 OR S11 OR S12 OR S13 | 34,977 |  |  |  |
| S13 | parent workshops | 589 |  |  |  |
| S12 | DE "Parent Influence" | 6,627 |  |  |  |
| S11 | DE "Parent Role" | 9,860 |  |  |  |
| S10 | DE "Parents" OR DE "Parents as Teachers" OR DE "Parenting Skills" OR DE "Parent Education" OR DE "Parent Counseling" | 20,578 |  |  |  |
| S9 | peer mediated intervention* | 98 |  |  |  |
| S8 | parent support* | 877 |  |  |  |
| S7 | parenting intervention* | 151 |  |  |  |
| S6 | parent mediated intervention* | 28 |  |  |  |
| S5 | parent intervention* | 94 |  |  |  |
| S4 | parent Training* | 7,186 |  |  |  |
| S3 | S1 OR S2 | 20,620 |  |  |  |
| S2 | DE "Pervasive Developmental Disorders" OR DE "Asperger Syndrome" OR DE "Autism" OR DE "Asperger Syndrome" OR DE "Developmental Disabilities" | 19,559 |  |  |  |
| S1 | Autism* or ASD | 16,078 |  |  |  |
