# Supplementary material for: Parent-Mediated Interventions for Children and Adolescents With Autism Spectrum Disorders: A Systematic Review and Meta-Analysis
Source: Front Psychiatry. 2021 Nov 12;12:773604. doi: 10.3389/fpsyt.2021.773604 (PMC8632873; doi:10.3389/fpsyt.2021.773604)
Supplement: Supplementary Table 3 — Characteristics of excluded studies. [file Table_3.DOCX]

**Table S3 List of excluded studies**

**Beaudoin A.J.; Sebire G.; Couture M.**

Parent-mediated intervention tends to improve parent-child engagement, and behavioral outcomes of toddlers with ASD-positive screening: A randomized crossover trial

Research in Autism Spectrum Disorders 2019;66(Journal Article):101416

| Reason for exclusion: |  | Wrong indication |
| --- | --- | --- |

**Bradshaw, Jessica; Bearss, Karen; McCracken, Courtney; Smith, Tristram; Johnson, Cynthia; Lecavalier, Luc; Swiezy, Naomi; Scahill, Lawrence**

Parent education for young children with autism and disruptive behavior: Response to active control treatment

Journal of Clinical Child and Adolescent Psychology 2018;47(Suppl 1):S445-S455

| Reason for exclusion: | Wrong comparator |
| --- | --- |

**Brignell, Amanda; Chenausky, Karen V.; Song, Huan; Zhu, Jianwei; Suo, Chen; Morgan, Angela T.**

Communication interventions for autism spectrum disorder in minimally verbal children

The Cochrane database of systematic reviews 2018;11(Journal Article):CD012324

| Reason for exclusion: | Wrong study design |
| --- | --- |

**Corti, Claudia; Pergolizzi, Francesca; Vanzin, Laura; Cargasacchi, Giulia; Villa, Laura; Pozzi, Marco; Molteni, Massimo**

Acceptance and Commitment Therapy-Oriented Parent-Training for Parents of Children with Autism

Journal of Child & Family Studies 2018;27(9):2887-2900

| Reason for exclusion: | Wrong study design |
| --- | --- |

**Dababnah S.; Olson E.M.; Nichols H.M.**

Feasibility of The Incredible Years Parent Program for Preschool Children on The Autism Spectrum in two U.S. sites

Research in Autism Spectrum Disorders 2019;57(Journal Article):120-131

| Reason for exclusion: | Wrong study design |
| --- | --- |

**Freitag, Christine M.; Jensen, Katrin; Teufel, Karoline; Luh, Marvin; Todorova, Antoaneta; Lalk, Christopher; Vllasaliu, Leonora**

[Empirically based developmental and behavioral intervention programs targeting the core symptoms and language development in toddlers and preschool children with autism spectrum disorder]

Zeitschrift fur Kinder- und Jugendpsychiatrie und Psychotherapie 2020;(Journal Article):1-18

| Reason for exclusion: | Wrong language |
| --- | --- |

**Gengoux, Grace W.; Schapp, Salena; Burton, Sarah; Ardel, Christina M.; Libove, Robin A.; Baldi, Gina; Berquist, Kari L.; Phillips, Jennifer M.; Hardan, Antonio Y.**

Effects of a parent-implemented Developmental Reciprocity Treatment Program for children with autism spectrum disorder

Autism: the international journal of research and practice 2019;23(3):713-725

| Reason for exclusion: | Wrong study design |
| --- | --- |

**Green, Jonathan; Pickles, Andrew; Pasco, Greg; Bedford, Rachael; Wan, Ming Wai; Elsabbagh, Mayada; Slonims, Vicky; Gliga, Teea; Jones, Emily; Cheung, Celeste; Charman, Tony; Johnson, Mark; British Autism Study of Infant Siblings (BASIS) Team**

Randomised trial of a parent-mediated intervention for infants at high risk for autism: Longitudinal outcomes to age 3 years

Journal of Child Psychology and Psychiatry 2017;58(12):1330-1340

| Reason for exclusion: | Wrong patient population |
| --- | --- |

**Heidlage, Jodi K.; Cunningham, Jennifer E.; Kaiser, Ann P.; Trivette, Carol M.; Barton, Erin E.; Frey, Jennifer R.; Roberts, Megan Y.**

The effects of parent-implemented language interventions on child linguistic outcomes: A meta-analysis.

Early Childhood Research Quarterly 2019;(Journal Article):

No Pagination Specified 2019

| Reason for exclusion: | Wrong study design |
| --- | --- |

**Ho M.-H.; Lin L.-Y.**

Efficacy of parent-training programs for preschool children with autism spectrum disorder: A randomized controlled trial

Research in Autism Spectrum Disorders 2020;71(Journal Article):101495

United Kingdom Elsevier Ltd 2020

| Reason for exclusion | Wrong comparator |
| --- | --- |

**Lecavalier, Luc; Smith, Tristram; Johnson, Cynthia; Bearss, Karen; Swiezy, Naomi; Aman, Michael G.; Sukhodolsky, Denis G.; Deng, Yanhong; Dziura, James; Scahill, Lawrence**

Moderators of parent training for disruptive behaviors in young children with autism spectrum disorder

Journal of abnormal child psychology 2017;45(6):1235-1245

| Reason for exclusion | Wrong comparator |
| --- | --- |

**Palmer, Melanie; Tarver, Joanne; Paris Perez, Juan; Cawthorne, Thomas; Romeo, Renee; Stringer, Dominic; Hallett, Victoria; Mueller, Joanne; Breese, Lauren; Hollett, Megan; Beresford, Bryony; Knapp, Martin; Slonims, Vicky; Pickles, Andrew; Simonoff, Emily; Scott, Stephen; Charman, Tony**

A novel group parenting intervention to reduce emotional and behavioural difficulties in young autistic children: protocol for the Autism Spectrum Treatment and Resilience pilot randomised controlled trial

BMJ open 2019;9(6):e029959

| Reason for exclusion | Wrong study design |
| --- | --- |

**Ruane, Ailbhe; Carr, Alan; Moffat, Valerie; Finn, Tania; Murphy, Angela; O'Brien, Orla; Groarke, Hilary; O'Dwyer, Roisin**

A randomised controlled trial of the Group Stepping Stones Triple P training programme for parents of children with developmental disabilities

Clinical Child Psychology & Psychiatry 2019;24(4):728-753

| Reason for exclusion: | Wrong patient population |
| --- | --- |

**Whitehouse A.J.O.; Varcin K.J.; Alvares G.A.; Barbaro J.; Bent C.; Boutrus M.; Chetcuti L.; Cooper M.N.; Clark A.; Davidson E.; Dimov S.; Dissanayake C.; Doyle J.; Grant M.; Iacono T.; Maybery M.; Pillar S.; Renton M.; Rowbottam C.; Sadka N.; Segal L.; Slonims V.; Taylor C.; Wakeling S.; Wan M.W.; Wray J.; Green J.; Hudry K.**

Pre-emptive intervention versus treatment as usual for infants showing early behavioural risk signs of autism spectrum disorder: a single-blind, randomised controlled trial

The Lancet Child and Adolescent Health 2019;3(9):605-615

| Reason for exclusion: | Wrong patient population |
| --- | --- |
