# Supplementary material for: Parent-Mediated Interventions for Children and Adolescents With Autism Spectrum Disorders: A Systematic Review and Meta-Analysis
Source: Front Psychiatry. 2021 Nov 12;12:773604. doi: 10.3389/fpsyt.2021.773604 (PMC8632873; doi:10.3389/fpsyt.2021.773604)
Supplement: Supplementary Table 4 — Characteristics of the included studies. [file Table_4.DOCX]

| **Table S4 Characteristics of Included Studies** | | | | | | | |  |  |  |  |
| --- | --- | --- | --- | --- | --- | --- | --- | --- | --- | --- | --- |
| **Study’s first Author, year, country** | **Age of children Mean active (control)** | **Intervention** | **Control group** | **Inclusion criteria** | **Length of intervention** | **Study design N (%male)** | **Race**  **/ethnicity** | **Socio-economic status** | **Reported outcomes** | **Overall results** | **Conflict of interest** |
| *Aldred, C. et al. (2004), UK* | 2-5 years  48 months (51 months) | Individually delivered social communication intervention. Parents are trained in shared attention, adaptive communication. | Treatment as usual | ADI criteria for classical autism | 6 months monthly sessions, 6 months bi-monthly sessions. | Pilot RCT, 24 (89%) | 2 mothers African-Caribbean, remaining Caucasian | Predominantly middle class, Mean education 17.6 years (16-21) fathers, 16.5 (16-21) years mothers | ADOS  VABS  PSI | Treatment group significant improvement on ADOS total. Suggestive, non-significant on VABS | Funded by a grant from the Shirley Foundation |
| *Aman, M. et al. (2009), USA* | 4-13 years 7.38 years (7.50 years) | Medication (risperidone) and individually delivered parent training.  Adaptive skills, social communication, visual communication, positive reinforcement, compliance. | Medication (risperidone) alone | Diagnosis of ASD established by DSM-IV criteria | Max. 17 sessions M=10.9 sessions | RCT, 124 (85%) | White 93, Hispanic 11, African American 18, Asian American 3, Native American 1 | Not reported | Parent-rated HSQ | HSQ showed an additive effect in the intervention group (p=0.006) | Funded by: Bristol-Meyers Squibb, Johnson & Johnson, Forest, consultant, research grant. Eli Lilly, CureMark, Neuropharm,  Novartis, Noven, Organon, Shire, Sigma Tau,  consultant, Targacept, Janssen, Supernus, Neuropharm, |
| *Barrett, A.C. et al. (2020), USA* | 1.5-4.5 years  35.75 months (38.22 months) | Individually delivered parent training; *Pivotal Response Intervention for Social Motivation (PRISM*). Behavioral reinforcement of child communication. Parent education. | Waiting list | Meeting ASD diagnostic DSM-V criteria | 6 months, *M*= 6.81 h. /week | Pilot RCT, 21 (90%) | White 57%, Latino 19%, Asian 9,5%, Multi-racial 9,5% | Not reported | No prespecified outcomes |  | Funded by Autism Speaks grant |
| *Bearss, K. et al., (2015*), USA | 3-7 years 4.8 (4.7) | Individually delivered parent training to manage disruptive behavior. Behavioral analysis, reinforcement of adaptive strategies, management of noncompliance. | 12 sessions parent education in autism, no management strategies. | ASD according to DSM-IV-TR criteria. >14 on ABC irritability subscale. Rating of moderate or higher on CGI-S | 12 sessions, 1 home visit, 2 optional sessions, 6 parent-child coaching sessions. 2 telephone booster sessions. Max 23 sessions. | RCT, 180 (87%) | White 156, Black 15, Asian 8, Other 1 | Maternal education active %(control%):  Advanced degree 32.6 (25.34), College 24.7 (40.7), Some college 31.5 (28.6), High school grad 10.1 (5.5), Some high school 1.1 (0) | Adaptive functioning: Parent-rated CGI  Conduct problems: HSQ | CGI improved 68.5% active versus 39.6% control (p<0.001)  HSQ declined 55% active versus 34.2% control (p<0.001) | Funded and supported by the National Institute of Mental Health |
| *Brian, J.A. et al., (2017),*  *Canada* | 16-30 months, 25.3 (25.19) | Home-based, individually delivered ”*Social ABCs”* (Pivotal Response Treatment principles). Behavioral, play-based reinforcement of child communication, functional communication. | Treatment as usual with restrictions of other parent interventions | Elevated scores on key ASD assessments supported by clinician impression regarding ASD symptoms | 12 weeks | RCT, 62 (76%) | White 48, Asian 7, Black 2, Mixed 5 | Parental education: High school 12, College/Trade 24, University 25, undisclosed 1 | VABS-II  ADOS-2  PSI (Parent Stress Index) | No significant between groups difference in any of the outcomes | Funded by Autism Speaks Canada, the Sinneave Family Foundation, and the Craig Foundation, with support from the Joan and Jack Craig Chair in Autism Research and the Stollery Child-ren´s Hospital Foundation Chair in Autism Research |
| *Carter, A.S. et al, 2011, USA* | 15-25 months, 20.25 months | Hanens *”More than words”* parent training program, group and individually. Focus on enhancing child communication, speech. | Treatment as usual | Meeting symptom criteria for ASD based on expert clinician evaluation | 3,5 months | RCT, 62 (82%) | White 44.7%, Hispanic/Latino 38.6%, Black 3.5%, Asian/White 5.3%, American Indian/Alaskan native /White 3.5% American Indian /Alaskan native /Hispanic 1.8%, | Parent education: High school 16%, Some college/ Associate degree /Trade degree 33%, College 35%, Advanced degree 16% | ADOS | No significance | Supported by a grant from Autism Speaks and the Marino Autism Research Institute. |
| *Dawson, G. et al., (2010) USA* | 18-30 months  23.9 (23.1) | Individually delivered ”*Early Start Denver Model” (ESDM)*, additional therapist training of child. Behavioral reinforcement of child social communication and language acquisition. Both parent training and in-home clinician coaching/training. | Treatment as usual + recommendations, referrals. | ASD diagnosis according to DSM-IV citeria | 2 years in total. Number of parent training sessions not reported | RCT, 48 (71%) | Asian 12.5%, White 72.9%, Latino 12.5%, Multi-racial 14,6 % | Not reported | VABS-II  ADOS-2 CSS | Significant improvement in adaptive behavior (Vineland) in intervention group compared to control. ADOS scores did not differ between groups- | This study was supported by National Institute of Mental Health |
| *Drew, A. et al., (2002), UK* | 21-36 months 21.4 (23.6) | Individually delivered social-pragmatic joint attention focused parent training. Reinforcement of joint attention, communication, language, management of disruptive behavior. | Treatment as usual | Meeting ICD-10 criteria for autism using the CHAT screening tool and ADI-R | 12 months, number of sessions not reported. | Pilot RCT, 24 (gender not reported) | Not reported | Not reported | Clinician rated ASD core symptoms ADI-R,  Parent stress  (PSI) | No significant between group difference in symptom severity (ADI-R) or parent stress (PSI) | A Medical Research Council Project Grant and a grant from the Special Trustees of Guys Hospital supported this  research. |
| *Gengoux, G.W. et al, (2019), USA* | 2-5 years 49.5 months (47.2 months) | Individually delivered *Pivotal Respons Treatment.*  Behavioral reinforcement of child social communication and language acquisition. Both parent training and in-home clinician coaching/training. | Delayed Treatment | ASD according to DSM-V criteria based on ADI-R + ADOS. And significant language delay. | 24 weeks. 15 sessions parent training + children trained by clinician at home. | RCT, 43 (88%) | White 28%, Asian American 56%, Hispanic 7%, Native Hawaiian 2%, bi-racial or other 7% | 84% college graduates | Clinician-rated symptom severity CGI  Parent-rated ASD core symptoms SRS-2 | CGI significant improvement in PRT group compared to control group. No significant between group effect on SRS-2 | Supported by the National Institute on Deafness and Other Communication Disorders, National Institute of Mental Health, Mentored Research Scientist Development Award Funded by the National Institutes of Health (NIH) |
| *Ginn, N.C et al., (2017), USA* | 3-7 years 4.32 (5.12) | Individually delivered *Child-Directed Interaction Training (CDIT*). Intervention target is procosial behaviors, increased positive parenting, reduction of disruptive behavior and parent stress. | Waiting list | Previous diagnoses of ASD from health care professional. Cognitive functioning >= 2 years | 8 sessions/ 10 weeks | RCT, 30 (80%) | 86.7 Caucasian | 80% of mothers completed >= 2 years of college | SRS-2  Eyberg Child Behavior Inventory (ECBI)  PSI | Significant improvement of child disruptive disorder (ECBI) and social awareness (SRS-2) in intervention group compared to control. No significance on PSI. | Supported by grants from the University of Florida Center for Pediatric Psychology and Family Studies |
| *Green, J. et al., (2010), UK* | 2-4 years 45 months (45 months) | Individually delivered *Preschool Autism Communication Trial (PACT).* Targets social interactive and communication impairments, increase parental responsiveness in order to decrease child impairment. Child+ parents trained. | Treatment as usual | Meeting criteria for core autism according to international diagnostic tests (ADOS-G and two of three domains of ADI-R) | 18 sessions/6 months. | RCT, 152 (92%) | White 55%, Mixed 12%, Non-white 33% | At least one parent in professional or administrative occupation: 66% in intervention group, 59% in control group | ADOS | No significant between group difference on ADOS total: Effect size -0.24 (95% CI  -0.59 – 0.11) | Sponsored by UK Medical Research Council and UK Department for children, Schools and Families |
| *Hardan, A. Y. et al., (2015), USA* | 2-6 years 4.1 (4.1) | Group *Pivotal Response Treatment.* Intervention targets behavioral reinforcement of child social communication and language acquisition. | 12 weeks of psychoeducation | Diagnosed with ASD based on DSM-IV criteria, ADI-R, ADOS and clinical judgement | 12 sessions/12 weeks | RCT, 53 (77%) | Not reported | M household income $125,000 - $150,000 range. 80% college graduates, 35% with graduate degrees | VABS  SRS  CGI-severity  Harms | VABS communication subscale significant improvement in treatment group compared to control (p=0.041) and CGI (p<0.001) No significance for SRS | Supported by: Autism Speaks Treatment Grant. Data manage-ment was supported by the National Center for Research Resources and the National Center for Advancing Transla-tional Sciences and National Institutes of Health |
| *Iadarola, S. et al., (2018), USA* | 3-7 years 4.8 (4.7) | Individually delivered parent training with a focus on reducing disruptive behavior, behavioral reinforcement and behavioral analysis. | 13 weeks of psychoeducation. No management strategies | ASD diagnosis based on DSM-IV-TR criteria. CGI-severity score >= 4, >= 15 on ABC irritability subscale | 16 weeks /15 sessions | RCT  180 (87,8%) | African-American 15, Asian 8, White/Caucasian 156, Other 1 | Family income: <$20,000 15, $20,001-40,000 37, $40,001-$60,000 36, $60,001-$90,000 36, >$90,000 56 | ABC irritability subscale  PSI | Not significant improvement on PSI compared to control. Significantly greater improvement of child disruptive behavior in intervention group compared to control (p<0.001) | Funded by the National Institute of Mental Health, and supported by: The National Center for Advancing Transla-tional Sciences of the National Institutes of Health and the Marcus Foundation |
| *Jocelyn, L.J. et al., (1998), Canada* | 24-72 months, 42.6 (43.8) | Day care + parent training with a focus on child social communication, functional analysis. | Day care alone | Diagnosis of pervasive developmental disorder DSM-III criteria. | 12 weeks | RCT, 35 (97%) | Caucasian 94%, Other 6% | Not reported | Parent- and clinician-rated ABC. SACC | No significance | Supported by Grant from the National Health Research and Develop-ment Program. |
| *Kasari, C. et al., (2010), USA* | 21-36 months 30.35 (31.31) | Individually delivered joint attention parent training. Focus on joint attention, play skills, behavioral reinforcement of child response. | Waiting list | Meeting criteria for autism following DSM-IV criteria by an independent clinician and no additional syndromes | 24 sessions/8 weeks | RCT, 38 (76%) | Caucasian 22, Minority 16 | Caregivers education: Some college 5, College 23, Graduate 10 | No prespecified outcomes |  | This study was supported by a NIMH grant |
| *Kuravackel, G.M. (2018), USA* | 3-12 years  TH 82.3 months, FF 104.6 months (101.8 months) | Group and individually delivered *COMPASS for Hope (C-HOPE)* parent training, content is behavioral analysis, psychoeducation, reinforcement of child response, supporting positive behavior. Delivered as telehealth (TH) or face to face (FF) | Waiting list | ASD diagnosis following DSM-IV/V and confirmed by ADOS-2 | 8 sessions | RCT, 33 (21%) | White 27, Black 5, Asian 1 | Household income in $: <10,000 1, 10,000-24,999 6, 25,000-49,999 10, 50,000-99,999 11, >100,000 3, missing 1 | ECBI  PSI | Significant pre- and post- test improvement in both child’s behavior (ECBI) and Parent stress (PSI) | Funding source: The University of Louisville and Uni-versity of Kentucky Collabora-tive Research initiative |
| *Lecavalier, L. et al., (2018), USA* | 5-14 years, 7.7 (8.2) | 1.Placebo + Individually delivered parent training. Parent training targets disruptive behavior.  2. Atomoxetine + PT | 3. Placebo  4. Atomoxetine | ASD diagnosis based on DSM-IV-TR criteria, clinical expert evaluation. Overactivity and/or inattention. CGI score >=4 | 10 weeks | RCT 2x2, 128 (85%) | Caucasian 105 (82%), African American 10 (8%) | Family income: >=$60,000 68 (53%), <$60,000 60 (47%) | PSI | PSI dropped for all groups but with no statistically significant differentiation between groups | AMO Pharma,  CogState, Inc.; Confluence Pharmaceu-tica; CogState Clinical Trials, Ltd.; Coronado Biosciences; Forest Research; Hoffman-La Roche; Johnson & Johnson, Lumos  Pharma, MedAvante, Inc.; Novartis; Ovid Therapeutics, ProPhase LLC; Supernus Pharmaceuticals, Lilly, Roche,  Curemark, Autism Speaks, NIMH, and NIA, Neuro-pharm, Noven, Shire,  Young Living, NIH, Gowlings, Pfizer, Sigma Tau, Tris Pharma, Waypoint. Arbor, Ironshore, Otsuka, Seaside Thera-peutics, Sunovion Pharmaceuticals, F.  Hoffman-La Roche AG. |
| *Pajareya, K., et al., 2011, Thailand* | 2-6 years, 56.6 months (55.1 months) | Individually delivered *DIR Floortime* parent training, social-pragmatic communication intervention. | Treatment as usual | ASD diagnosis according to DSM-IV criteria, confirmed by developmental pediatrician | 15 hours, Three months | Pilot RCT, 32 (87.5%) | Not reported | 75% of mothers has bachelor´s degree or higher | Parent-rated ASD core symptoms Childhood Autism Rating Scale (CARS) | Significant reduction of parent-reported symptoms on CARS (p=0.002) | Not reported |
| *Reitzel, J., et al., (2013), Canada* | 38-84 months, 57.9 months (62.6 months) | Group based *Functional behavior Skills Training* with parents and children. Focus on functional play, reciprocal communication, reinforcement of adaptive behavior. | Treatment as usual | diagnosis of autistic disorder by a physician based on  the DSM-IV-TR, ADOS or ADI-R that meet classification for ASD, and being unable to perform 80% of items on the Early Learning Measure  pretest | 2 hours weekly sessions/ 4 months. | RCT, 15 (Not reported) | Not reported | Not reported | VABS Parent Sense of Competence (PSOC) | No significant between group difference on VABS or PSOC | Funding provided by the Ontario Mental Health Foundation |
| *Schertz, H.H. et al, 2013, USA* | < 30 months,  *24.6 (27.5)*  months | *Joint attention mediated learning (JAML*) parent training with video-based feedback. Focus on joint attention, turn taking and focusing on faces. | Treatment as usual | Scores above cutoff on ADOS and absence of joint attention in observation of play session with parent | 4-12 months, *M* ~ 7 months | RCT, 23 (Not reported) | Not reported | Parent education (Years M/SD) Intervention 14.4/2.3 Control 15.8/2.3 | VABS | Significant change of adaptive functioning in intervention group and not in control group | Supported by a grant from Autism Speaks |
| *Schertz, H.H. et al, 2018, USA* | 16-30 months  24.55 (24.79) | *Joint attention mediated learning (JAML*) parent training with video- based feedback. Focus on joint attention, turn taking and focusing on faces. | Community control | Scores above cutoff on ADOS-T and < 3 instances of joint attention in 10-minute play session | 1 h sessions weekly/32 weeks | RCT, 144 (79.45) | White 77% (70%), Black 21% (18%), Hispanic 16% (10%), Other 14% (21%) | Parent income: <$19,091 26, $19,091-$30,971 10, $30,971-$40,000 11, $40,000-$59,000 29, >$60,000 63 | No prespecified outcome | No prespecified outcome | Support from the Institute for Education  Sciences, U.S. Department of Education, and a grant from the Institute for Education Sciences,  U.S. Department of Education |
| *Siller, M. et al.,( 2013), USA* | 32-82 months, 55.3 (58.3) | Individually delivered *Focused Playtime Intervention.* Parent training targeting parental responsiveness to child’s communication, reinforcement of child initiatives, play+ Parent education. | Control group (including parent education) | Diagnosed with ASD and meeting diagnostic DSM-IV criteria on ADI-R | 12 weeks | RCT, 70 (not reported) | Hispanic 31, White 14, Asian 13, Black 5, Mixed 7 | Family income: Below $19,999 8, $20,000-$39,999 13, $40,000-$74,999 17, >$74,999 32 | No prespecified outcome | No prespecified outcome | Supported by CPEA Grant from the National Institute of Child Health and Human Development, the  M.I.N.D. Institute Research Program, and a PSC-CUNY grant. |
| *Solomon, M. et al., (2008), USA* | 5-12 years, 8.2 (8.1) | Individually delivered *Parent Child Interaction Therapy.* Targets disruptive behavior and reinforcement of positive behaviour. | Waiting list | ASD diagnosis following DSM-IV-TR criteria and ADOS-G and ADI-R  IQ>70 and significant externalizing behavior on BASC | 13 sessions | Pilot RCT, 19 (100%) | Not reported | Not reported | ECBI  PSI/parent stress subscale | Parents in both groups reported declines in ECBI scores. Not significantly more in intervention group. No significant change on PSI. | Supported by a Children´s Miracle Network Grant from the U.C. Davis Medical Center |
| *Solomon, R. et al., (2014), USA* | 2-6 years, 49.85 months (50.53 months) | Individually delivered home-based *PLAY* intervention, focus on enhancing child development and social communication through play. | Treatment as usual | ASD according to DSM-IV criteria, and meeting criteria for ASD on ADOS and SCQ | 3 h months/12 months | RCT, 128 (82%) | Child of color: 18.8% intervention, (29.7% control group) | Family income <$60.000 54.0% intervention (56.3% control group) | Parent- rated Autism core symptoms on Social Communication Questionnaire (SCQ)  PSI | SCQ improved over time but with no significant between group difference. Parent stress decreased but no significant between group difference. | Funded by National Institute of Mental Health (NIMH) and Small Business Innovation Research (SBIR) grant |
| *Tonge, B. et al., (2014), Australia* | 23-70 months, 46 (50.11) | Group-and individually delivered *Preschool with Autism Parent* training programme, cognitive-behavioral parent training with focus on psychoeducation, behavioral reinforcement of child behavior and social communication, parent support. | Treatment as usual | Diagnosed with pervasive developmental disorder at two metropolitan and two rural autism assessment services. | 20 weeks | RCT, 107 (Not reported) | Ethnic background: British 7, Irish 1, Italian 5, Greek 8, Vietnamese 2, Chinese 1, Cambodian 1, Sudanese 1, Saudi Arabian 1, Indian 2, Pacific Icelander 1, Indigenous Australian 1 | Professional 50, Business management 5, farming 6, trades 20 unskilled or semi-skilled workers 14, unemployed 8 | VABS daily living  CARS | A significant difference in favor of the intervention on VABS daily living. Superiority of the individualized treatment compared to the group treatment. No significant between group difference on CARS | Supported by NH&MRC of Australia Project  Grant. |
| *Valeri, G. et al., (2019); Italy* | 2-11 years, 4.4 (4.2) | Individually based *Cooperative parent-mediated therapy*. Focus on social communication, emotion regulation, joint attention, cognitive flexibility, imitation. Administered in addition to low intensity psychosocial intervention (TEACCH based). | Low intensity psychosocial intervention (TEACCH-based) | Meeting ASD criteria on DSM-IV-TR and over cutoff for ASD on ADOS-G and ADI-R | 15 sessions/6 months | RCT, 34 (79%) | Not reported | Education: Middle/Elementary school 16%, High School 37%, University 47% | ADOS-2 CSS, CBCL-EXT,  PSI | Significant decrease of ASD symptoms on ADOS-G in intervention group compared to control (p=0.013). And decrease of parent stress in favor of intervention on PSI (p=0.018) No significance on CBCL-EXT | Not reported |
| *Vernon, T. W., et al, (2019), USA* | 1.5-4.5 years, 35.75 months (34.45 months) | Individually delivered *Pivotal Response Intervention for Social Motivation* (PRISM). Behavioral reinforcement of child communication and social response. Parent education. | Waiting list | ASD diagnosis based on DSM-V and over cut-off for autism on ADOS | 10 h/week, 26 weeks | Pilot RCT, 23 (87%) | White 13, Latino 5, Asian 3, Multi-racial 2 | Not reported | VABS  ADOS-2 CSS | Significant improvement of ASD core symptoms on ADOS-2 CSS and adaptive functioning on VABS communication subscale. | This study was funded by Autism Speaks |
| *Wittingham, K., et al., (2009), Australia.* | 2-9 years, 6.62 (6.20) | Group-and individually based *Stepping Stones Tripple P* parenting programme. Focus on parent management of child behavior, parenting strategies. | Waiting list | ASD diagnosis from paediatrician and clinical DSM-IV verification | 8 weeks | RCT, 59 (79.7%) | Not reported | Not reported | ECBI,  Being a Parent Scale Satisfaction | Significant decrease of disruptive behavior on ECBI in intervention compared to control group. Suggestive but not significant effect of intervention of Being a Parent Scale Satisfaction | Supported by a small grant from the School of Psychology, The University of Queensland. |
| *Williams, M.E. et al, (2020), UK* | 3-8 years, 68.03 months (67.93 months) | Group-based *Incredible Years* *Autism Spectrum and Language Delay (IY-ASLD*. Focus on parent-child relationship, child development, social, emotional and adaptive skills. | Waiting list | Recently diagnosed or strongly suspected of ASD | 2 h/week, 12 weeks | RCT, 58 (71%) |  | Parent education: <17 years left school 55%, >=17 years left school 45%. No employment in household 21%; Employment in household 79% | CBCL-EXT,  PSI | No significance | funded by Autistica |
| *Wong, C.C.N., et al., (2010), China* | 17-36 months, 25.33 (27.88) | Individually based parent training in *Autism 1-2-3. F*ocus on eye contact, gestures and communication. | No intervention control | Diagnosed with ASD using DSM-IV criteria, ADOS and ADI-R | 5 days/week, 2 weeks | RCT, 17 (94%) | Not reported | Not reported | Parent distress subscale narratively described | No significant between group difference of parent stress | Not reported |

## List of abbreviations

ADOS: Autism Diagnostic Observation Schedule

ADOS-2 CSS: Autism Diagnostic Observation Schedule 2^nd^ Edition Calibrated Severity Score

VABS: Vineland Adaptive Behavior Scale

PSI: Parent Stress Index

HSQ: Home Situations Questionnaire

CGI: Clinical Global Impressions Scale

ADI-R: Autism Diagnostic Interview - Revised

SRS-2: Social Responsiveness Scale 2^nd^ Edition

ECBI: Eyberg Child Behavior Inventory

ABC: Aberrant Behavior Checklist

CARS: Child Autism Rating Scale

PSOC: Parent Sense of Competence

SCQ: Social Communication Questionnaire

CBCL: Child Behavior Checklist
