# Supplementary material for: Parent-Mediated Interventions for Children and Adolescents With Autism Spectrum Disorders: A Systematic Review and Meta-Analysis
Source: Front Psychiatry. 2021 Nov 12;12:773604. doi: 10.3389/fpsyt.2021.773604 (PMC8632873; doi:10.3389/fpsyt.2021.773604)
Supplement: Supplementary Table 5 — Summary of findings table. [file Table_5.DOCX]

| **Table S5. Summary of findings:** | | | |
| --- | --- | --- | --- |
| Parent-mediated interventions for children and adolescents with autism spectrum disorders: a systematic review and meta-analysis | | | |
| **Patient or population**: Children with autism spectrum disorders aged 2-17 years  **Intervention**: Parent-mediated interventions  **Comparison**: No parent-mediated intervention | | | |
| Outcomes | Relative effect  (95% CI) | No. of participants  (Studies) | Certainty of the evidence  (GRADE) |
| Primary: Adaptive functioning (parent-rated) (lower is better) Follow up: 4-24 months** | SMD: 0.28  (-0.01 to 0.57) | 301 (8 RCTs) ^(1-8)^ | ⨁⨁◯◯ LOW ^a,b^ |
| Primary: Adaptive functioning (clinician-rated) assessed with: CGI-severity (lower is better) Follow up: 3-24 months | SMD: -0.45  (-0.87 to -0.03) | 90 (2 RCTs) ^(9, 10)^ | ⨁◯◯◯ VERY LOW ^a,c^ |
| Secondary: Adverse Effects (lower is better) Follow up: 3-24 months | 0 less of 1000  (-50 to 50) | 98 (2 RCTs) ^(4, 10)^ | ⨁⨁◯◯ LOW ^c^ |
| Secondary: Autism Core Symptoms (parent-rated) (lower is better) Follow up: 2-6 months | SMD: 0.06  (-0.18 to 0.30) | 371 (7 RCTs) ^(7, 9-14)^ | ⨁⨁◯◯ LOW ^a,b^ |
| Secondary: Autism Core Symptoms (clinician-rated) (lower is better) Follow up: 3-6 months | SMD: -0.35  (-0.71 to 0.02 higher) | 456  (9 RCTs) ^(1-4, 8, 13, 15-17)^ | ⨁⨁◯◯ LOW ^a,b^ |
| Secondary: Disruptive behavior (parent-rated) (lower is better) Follow up: 2-6 months | SMD: -0.55  (-0.74 to -0.36) | 711 (9 RCTs) ^(12, 17-24)^ | ⨁⨁⨁◯ MODERATE ^a^ |
| Secondary: Parental well-being (lower is better) Follow up: 2-12 months | SMD: -0.16  (-0.32 to -0.01) | 699 (12 RCTs) ^(2, 5, 11-14, 17, 20, 21, 23-25)^ | ⨁⨁◯◯ LOW ^a,b^ |
| Secondary: Child anxiety  Follow up: minimum of 8 weeks |  | 0 (0) |  |
| Secondary: Quality of life of the child (parent-rated)  Follow up: minimum of 8 weeks |  | 0 (0) |  |
| ***The risk in the intervention group** (and its 95% confidence interval) is based on the assumed risk in the comparison group and the **relative effect** of the intervention (and its 95% CI).  ****** Adaptive functioning (parent-rated) at a minimum of eight weeks and minimum of six months synthesized as one outcome. **CI:** Confidence interval; **SMD:** Standardized mean difference; **MD:** Mean difference; **PMI**: Parent-mediated interventions; **CGI**: Clinical Global Impressions | | | |
| **GRADE Working Group grades of evidence** **High certainty:** We are very confident that the true effect lies close to that of the estimate of the effect **Moderate certainty:** We are moderately confident in the effect estimate: The true effect is likely to be close to the estimate of the effect, but there is a possibility that it is substantially different **Low certainty:** Our confidence in the effect estimate is limited: The true effect may be substantially different from the estimate of the effect **Very low certainty:** We have very little confidence in the effect estimate: The true effect is likely to be substantially different from the estimate of effect | | | |

a. Downgrading by one level due to risk of bias (lack of blinding of participants and outcomes assessors)

b. Downgrading one level due to Imprecision of the results (few participating children and wide confidence intervals)

c. Downgrading by two levels due to Imprecision of the results (few participating children, few studies and wide confidence intervals)

## References

1. Aldred C, Green J, Adams C. A new social communication intervention for children with autism: pilot randomised controlled treatment study suggesting effectiveness. Journal of Child Psychology and Psychiatry. 2004;45(8):1420-30.

2. Brian JA, Smith IM, Zwaigenbaum L, Bryson SE. Cross-site randomized control trial of the Social ABCs caregiver-mediated intervention for toddlers with autism spectrum disorder. Autism Res. 2017;10(10):1700-11.

3. Carter AS, Messinger DS, Stone WL, Celimli S, Nahmias AS, Yoder P. A randomized controlled trial of Hanen's 'More Than Words' in toddlers with early autism symptoms. J Child Psychol Psychiatry. 2011;52(7):741-52.

4. Dawson G, Rogers S, Munson J, Smith M, Winter J, Greenson J, et al. Randomized, Controlled Trial of an Intervention for Toddlers With Autism: The Early Start Denver Model. Pediatrics. 2009;125(1):e17-e23.

5. Reitzel J, Summers J, Lorv B, Szatmari P, Zwaigenbaum L, Georgiades S, et al. Pilot randomized controlled trial of a Functional Behavior Skills Training program for young children with Autism Spectrum Disorder who have significant early learning skill impairments and their families. Research in Autism Spectrum Disorders. 2013;7(11):1418-32.

6. Schertz HH, Odom SL, Baggett KM, Sideris JH. Effects of Joint Attention Mediated Learning for toddlers with autism spectrum disorders: An initial randomized controlled study. Early Childhood Research Quarterly. 2013;28(2):249-58.

7. Tonge B, Brereton A, Kiomall M, Mackinnon A, Rinehart NJ. A randomised group comparison controlled trial of 'preschoolers with autism': a parent education and skills training intervention for young children with autistic disorder. Autism. 2014;18(2):166-77.

8. Vernon TW, Holden AN, Barrett AC, Bradshaw J, Ko JA, McGarry ES, et al. A Pilot Randomized Clinical Trial of an Enhanced Pivotal Response Treatment Approach for Young Children with Autism: The PRISM Model. J Autism Dev Disord. 2019;49(6):2358-73.

9. Gengoux GW, Abrams DA, Schuck R, Millan ME, Libove R, Ardel CM, et al. A Pivotal Response Treatment Package for Children With Autism Spectrum Disorder: An RCT. Pediatrics. 2019;144(3).

10. Hardan AY, Gengoux GW, Berquist KL, Libove RA, Ardel CM, Phillips J, et al. A randomized controlled trial of Pivotal Response Treatment Group for parents of children with autism. J Child Psychol Psychiatry. 2015;56(8):884-92.

11. Drew A, Baird G, Baron-Cohen S, Cox A, Slonims V, Wheelwright S, et al. A pilot randomised control trial of a parent training intervention for pre-school children with autism. Preliminary findings and methodological challenges. Eur Child Adolesc Psychiatry. 2002;11(6):266-72.

12. Ginn NC, Clionsky LN, Eyberg SM, Warner-Metzger C, Abner JP. Child-Directed Interaction Training for Young Children With Autism Spectrum Disorders: Parent and Child Outcomes. J Clin Child Adolesc Psychol. 2017;46(1):101-9.

13. Jocelyn LJ, Casiro OG, Beattie D, Bow J, Kneisz J. Treatment of children with autism: a randomized controlled trial to evaluate a caregiver-based intervention program in community day-care centers. J Dev Behav Pediatr. 1998;19(5):326-34.

14. Solomon R, Van Egeren LA, Mahoney G, Quon Huber MS, Zimmerman P. PLAY Project Home Consultation Intervention Program for Young Children With Autism Spectrum Disorders: A Randomized Controlled Trial. Journal of Developmental & Behavioral Pediatrics. 2014;35(8):475-85.

15. Green J, Charman T, McConachie H, Aldred C, Slonims V, Howlin P, et al. Parent-mediated communication-focused treatment in children with autism (PACT): a randomised controlled trial. The Lancet. 2010;375(9732):2152-60.

16. Pajareya K, Nopmaneejumruslers K. A pilot randomized controlled trial of DIR/Floortime parent training intervention for pre-school children with autistic spectrum disorders. Autism. 2011;15(5):563-77.

17. Valeri G, Casula L, Menghini D, Amendola FA, Napoli E, Pasqualetti P, et al. Cooperative parent-mediated therapy for Italian preschool children with autism spectrum disorder: a randomized controlled trial. Eur Child Adolesc Psychiatry. 2020;29(7):935-46.

18. Aman MG, McDougle CJ, Scahill L, Handen B, Arnold LE, Johnson C, et al. Medication and parent training in children with pervasive developmental disorders and serious behavior problems: results from a randomized clinical trial. J Am Acad Child Adolesc Psychiatry. 2009;48(12):1143-54.

19. Bearss K, Johnson C, Smith T, Lecavalier L, Swiezy N, Aman M, et al. Effect of parent training vs parent education on behavioral problems in children with autism spectrum disorder: a randomized clinical trial. JAMA. 2015;313(15):1524-33.

20. Iadarola S, Levato L, Harrison B, Smith T, Lecavalier L, Johnson C, et al. Teaching Parents Behavioral Strategies for Autism Spectrum Disorder (ASD): Effects on Stress, Strain, and Competence. J Autism Dev Disord. 2018;48(4):1031-40.

21. Kuravackel GM, Ruble LA, Reese RJ, Ables AP, Rodgers AD, Toland MD. COMPASS for Hope: Evaluating the Effectiveness of a Parent Training and Support Program for Children with ASD. J Autism Dev Disord. 2018;48(2):404-16.

22. Solomon M, Ono M, Timmer S, Goodlin-Jones B. The effectiveness of parent-child interaction therapy for families of children on the autism spectrum. J Autism Dev Disord. 2008;38(9):1767-76.

23. Whittingham K, Sofronoff K, Sheffield J, Sanders MR. Stepping Stones Triple P: an RCT of a parenting program with parents of a child diagnosed with an autism spectrum disorder. J Abnorm Child Psychol. 2009;37(4):469-80.

24. Williams ME, Hastings RP, Hutchings J. The Incredible Years Autism Spectrum and Language Delays Parent Program: A Pragmatic, Feasibility Randomized Controlled Trial. Autism Res. 2020;13(6):1011-22.

25. Lecavalier L, Pan X, Smith T, Handen BL, Arnold LE, Silverman L, et al. Parent Stress in a Randomized Clinical Trial of Atomoxetine and Parent Training for Children with Autism Spectrum Disorder. J Autism Dev Disord. 2018;48(4):980-7.
