# Supplementary material for: Parent-Mediated Interventions for Children and Adolescents With Autism Spectrum Disorders: A Systematic Review and Meta-Analysis
Source: Front Psychiatry. 2021 Nov 12;12:773604. doi: 10.3389/fpsyt.2021.773604 (PMC8632873; doi:10.3389/fpsyt.2021.773604)
Supplement: Supplementary Table 6 — Characteristics of post-hoc identified studies. [file Table_6.DOCX]

| **Table S6 Characteristics of posthoc identified studies** | | | | | | | | | | | |
| --- | --- | --- | --- | --- | --- | --- | --- | --- | --- | --- | --- |
| **Study’s first Author, year, country** | **Age of children Mean active (control)** | **Intervention** | **Control group** | **Inclusion criteria** | **Length of intervention** | **Study design N (%male)** | **Race**  **/ethnicity** | **Socio-economic status** | **Reported outcomes** | **Overall results** | **Conflict of interest** |
| *Mahoney & Solomon (2016),, USA* | 2-6 years 49.85 months (50.53 months) | Individually delivered home-based PLAY inter-vention, focus on enhancing child development and social communi-cation through play. | Treatment as usual | ASD according to DSM-IV criteria, and meeting criteria for ASD on ADOS and SCQ | 3 hours months for 12 months | RCT 128 (82%) | Child of color: 18.8% intervention (29.7% control group) | Family income <$60.000 54.0% intervention (56.3% control group) | Autism Diagnostic Observation Schedule 2 (ADOS-2) Calibrated Severity Score (CSS) | Treatment group significant improvement on ADOS-2 Social Affect, but not restric-tive and Repetitive Behaviors, and no significance on ADOS CSS total. | Supported by National Institute of mental Health and Small Business Innovation Research grant. |
| *Rahman. et al. (2016), India and Pakistan* | 2-9 years 63.72 months (66.67 months) | Paediatric Autism Communication Therapy (PACT) culturally adapted for South Asia. Developmental intervention targets social communi-cation. | Treatment as usual | Meeting criteria for autism on INCLEN Diagnostic tool for Autism Spectrum Disorders | 12 sessions for 6 months | RCT, 65 (82%) | Not reported | Fathers education: Non-graduate 58%, Graduate 38%, Missing 3% | Vineland Adaptive Behavior Scale (VABS) | No significant result on VABS. No adverse events reported | Funded by Autism Speaks, USA |
| *Scahill et al. (2012), USA* | 4-13 years  7.50 years (7.38 years) | Medication (risperidone) and Parent training targeting mal-adaptive behaviors. Promoting compli-ance, functional communication, daily living skills and generalization of skills across time and place. | Medication alone (risperidone) | Meeting ASD diag-nostic DSM-IV TR criteria based on clinical assessment and ADI-R. And serious behavioral problems ABC-I >18 | 11 core sessions, one home visit and up to 3 optional sessions during first 16 weeks. Four booster sessions during week 16-24 (two telephone, one in clinic and a home visit) | RCT, 124 (85%) | White 75%, Hispanic 9%, African American 13%, Asian American 2%, Native American 1% | Not reported | Vineland Adaptive Behavior Scale (VABS) | Greater improvement on VABS in group with Parent training. Between group difference not significant when adjusted for IQ | Dr. Scahill serves as consultant for Biomarin, Boehringer-Ingelheim, Hoffman, Neuro-Search, and Pfizer |
