# Supplementary material for: Parent-Mediated Interventions for Children and Adolescents With Autism Spectrum Disorders: A Systematic Review and Meta-Analysis
Source: Front Psychiatry. 2021 Nov 12;12:773604. doi: 10.3389/fpsyt.2021.773604 (PMC8632873; doi:10.3389/fpsyt.2021.773604)

# Table S7. POST HOC ANALYSES

Forest plot of comparison: 2 Parent-mediated intervention vs no parent-mediated intervention post hoc, outcome: 2.1 Adaptive functioning, parent-rated, lower is better.


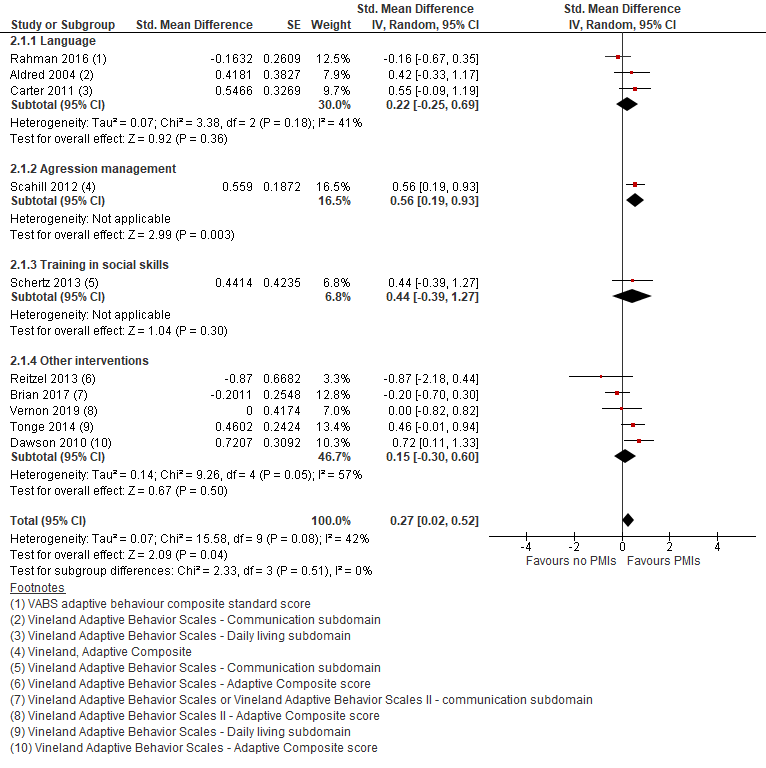


Forest plot of comparison: 2 Parent-mediated intervention vs no parent-mediated intervention post hoc, outcome: 2.5 Autism core-symptoms, clinician-rated.
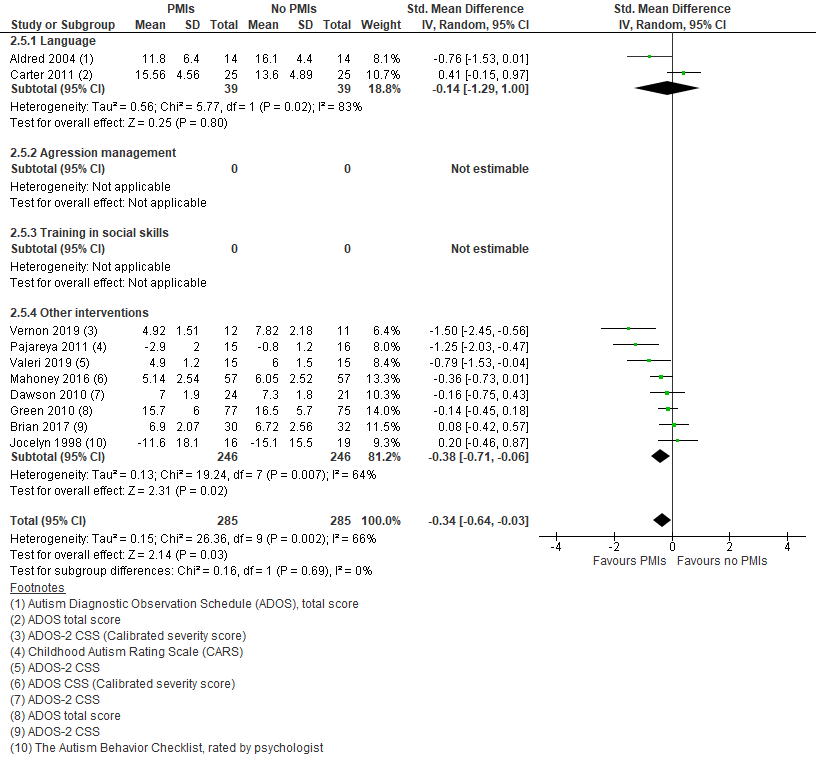

Supplement: Supplementary Table 7 — post-hoc analyses. [file Table_7.docx]
